# Rate-dependent inelastic deformation of Slochteren sandstone: implication for reservoir compaction in the Groningen gas field

**DOI:** 10.1007/s40948-025-01014-5

**Published:** 2025-07-15

**Authors:** Takahiro Shinohara, Berend A. Verberne, Christopher J. Spiers, Johannes. H. P. de Bresser, Suzanne J. T. Hangx

**Affiliations:** 1 Department of Earth Sciences, Utrecht University, Princetonlaan 4, 3584CD Utrecht, Netherlands; 2 Now at Department of Geoscience and Engineering, Delft University of Technology, Stevinweg 1, 2628 CN Delft, Netherlands; 3 Shell Global Solutions International B.V., Grasweg 31, 1031HW Amsterdam, Netherlands

**Keywords:** Uniaxial strain, Time-dependent, Stress–strain behaviour, Stress corrosion cracking, Grain rearrangement, Fluid extraction

## Abstract

Hydrocarbon production from sandstone reservoirs causes elastic and inelastic reservoir compaction, potentially leading to surface subsidence and even seismicity, such as observed in the Groningen gas field, Netherlands. Inelastic compaction can partly be instantaneous, though rate-/time-dependent processes may play a role on the longer term. Therefore, compaction may continue even if production is stopped. To reliably evaluate the impact of post-abandonment behaviour, mechanism-based rate-/time-dependent compaction laws are needed. We performed triaxial compression experiments on Slochteren sandstone (reservoir of the Groningen field) samples, with porosity $\phi =$ 14.6–18.9%, to investigate the effect of strain rate (rates of $10^{ - 6}{-}10^{ - 8} \,{\text{s}}^{ - 1}$) under conventional triaxial and uniaxial strain (i.e. zero-lateral strain) boundary conditions. Under triaxial conditions, lowering of stress–strain curves was observed with decreasing strain rate at all differential stresses, the effect being enhanced at higher temperature and pore fluid pH. By contrast, strain rate had limited effect on axial stress vs. strain behaviour under uniaxial strain conditions, though decreasing strain rate, as well as increasing fluid pH, resulted in a smaller increase in confining pressure required to maintain a zero-displacement lateral boundary condition. The mechanical data, complemented by microstructural analysis, suggest that subcritical cracking, coupled with grain rearrangement, was the dominant mechanism causing inelastic deformation under triaxial and uniaxial strain conditions. Our results suggest that the amount of reservoir compaction will be limited after production stops. However, time-dependent deformation will lead to changes in the in-situ state of stress, which should be included in models assessing reservoir compaction and induced seismicity in the Groningen field.

## Article Highlights

- Time-dependent inelastic compaction of Groningen reservoir sandstone at in-situ stress–strain conditions
- Limited grain rearrangement leads to negligible contribution of grain cracking to strain in uniaxial strain condition
- The amount of reservoir compaction in the Groningen field may be more limited than those predicted by conventional triaxial experiments

## Introduction

Prolonged extraction of hydrocarbons from subsurface sandstone reservoirs frequently leads to surface subsidence and is occasionally accompanied by induced seismicity (Davies et al. 2013; Fialko and Simons 2000; Hettema et al. 2002; Morton et al. 2001; Suckale 2009; Van Wees et al. 2014). The main cause for these two phenomena is an increase in effective stress due to reduction of pore pressure and subsequent compaction of the reservoir. Though the vertical reservoir strain is generally small, typically a few tenths of a percent up to 0.5%, the resulting surface subsidence can amount to several tens of centimeters, which may pose issues for the surface environment (Morton et al. 2001; Van Thienen-Visser and Breunese 2015). A prime example is the giant Groningen gas field in the densely populated north of the Netherlands. This has attracted much attention in recent years as surface subsidence has reached up to ~ 40 cm in the centre of the field (reservoir thickness ~ 200 m, NAM 2021), while induced seismicity has increased in frequency and magnitude since the 1990s (Van Thienen-Visser and Breunese 2015), declining again since reduced production in 2014. The largest earthquake to date, with a magnitude of 3.6, occurred in 2012 near Huizinge, located in the central part of the field. It’s here that the gas-bearing reservoir rock, the Slochteren sandstone, is most porous (18–24%; NAM 2013), and in-situ measurements suggest that 0.15–0.3% strain has occurred at the reservoir level to date (Cannon and Kole 2017). Since the reservoir is heavily faulted, with to date 1700 identified faults (NAM 2013), differential compaction across these faults frequently leads to induced seismicity (Buijze et al. 2017, 2019; Nagelhout and Roest 1997; Orlic and Wassing 2013; Roest and Kuilman 1994; Van Eijs et al. 2006). Furthermore, from nearby smaller fields, it is evident that compaction, and hence the drive for surface subsidence and induced seismicity, may continue (Ketelaar et al. 2011) even now that production is halted (Ministry of Economic Affairs and Climate Policy 2024).

Ongoing compaction of the reservoir after production is stopped would suggest that inelastic, time-dependent processes may play a role. Reversible, time-insensitive poroelastic compaction, which is often assumed to be controlling reservoir deformation (Bourne and Oates 2020; Dempsey and Suckale 2017; Laurent et al. 1993; Segall 1992; Van Eijs et al. 2006) can easily be quantified (H. Wang 2017). However, observations on highly porous sandstones in experiments (Bernabé et al. 1994; Hol et al. 2015, 2018; Pijnenburg et al. 2018, 2019a, 2019b; Shalev et al. 2014; Shinohara et al. 2024) and the field (Santarelli et al. 1998) have shown that significant permanent compaction can occur even at stresses well below the yield point identified in the stress–strain curve (i.e. at small strains of $<0.5\%$). For the Slochteren sandstone, under realistic stresses and strains, relevant to the depleting field, inelastic compaction accumulated during the production phase (i.e. from 1963 until 2024) was largely accommodated by virtually time- or rate-insensitive compaction of and slip along intergranular clay films (Pijnenburg et al. 2019b; Verberne et al. 2021). Now that production has stopped in April 2024 (Ministry of Economic Affairs and Climate Policy 2024), compaction may continue for some time to come, suggesting that time-dependent mechanisms may play a role (Brantut et al. 2014; De Waal and Smits 1988; Heap et al. 2015; Pijnenburg et al. 2018; Stanchits et al. 2009; Z. Wang et al. 2022).

Recent conventional triaxial experiments on Bleurswiller sandstone, an analogue material having petrophysical and mechanical properties comparable to the Slochteren sandstone, indeed observed time-dependent deformation at small stresses and vertical strains (~ 0.4%), and strain rates down to 10^–9^ s^−1^ (Shinohara et al. 2024). The main deformation mechanisms controlling rate-sensitive inelastic deformation of the Bleurswiller sandstone were frictionally controlled grain boundary sliding (Bernabé and Brace 1990; Menéndez et al. 1996; Pijnenburg et al. 2018; Shalev et al. 2014) coupled with an increase in the contribution of inter- or intragranular subcritical cracking at higher stresses (Brantut et al. 2013, 2014; Heap et al. 2009a, b; Heap et al. 2015; Pijnenburg et al. 2018). Extrapolation of the Bleurswiller data to a typical field strain rate of ~${10}^{-12} \, {\text{s}}^{-1}$ (i.e. similar to the field strain rate of the Groningen gas field; NAM 2021) using existing empirical models (Brantut et al. 2014; De Waal and Smits 1988; Pruiksma et al. 2015; Shinohara et al. 2024) suggests that additional strains of about 10% can be expected, compared to the strain accumulated at laboratory strain rates of $10^{ - 8}{-}10^{ - 9} \,{\text{s}}^{ - 1}$. Note that these strains are the slowest that currently can be achieved in laboratory experiments and come closest to simulating the very slow field strain rates of ~${10}^{-12} \, {\text{s}}^{-1}$ (Shinohara et al. 2024). However, it remains unclear whether extrapolation of the results from these experiments on analogue material, obtained under conventional triaxial boundary conditions, actually holds for the field, where the Slochteren sandstone is likely compacted under uniaxial strain boundary conditions (i.e. vertical strain but no lateral strain) (e.g. Dudley et al. 2016). This may have important consequences for the interaction between intergranular sliding and subcritical/stress corrosion crack growth. Furthermore, at lower stress levels and smaller strains (< 0.5%), relevant for depleting reservoirs, it is still unclear whether stress corrosion cracking can result in non-negligible amount of deformation, as there is a limited report that stress corrosion cracking is measurable at strain rates below 10^–8^ s^−1^, and smaller strains (< 0.5%) (Heap et al. 2015; Pijnenburg et al. 2018). To reliably predict subsidence and seismicity in the Groningen gas field, it is crucial to identify and quantify the grain-scale mechanisms leading to compaction in the Slochteren sandstone under realistic in-situ stress and boundary conditions.

In this study, we aim to quantify the additional contribution to inelastic compaction by time- or rate-dependent deformation mechanisms. This additional strain will be quantified compared to strain obtained during deformation at a typical laboratory strain rate of ${10}^{-6} \, {\text{s}}^{-1}$, under experimental conditions corresponding to in-situ reservoir pressure–temperature conditions and different boundary conditions to systematically identify the deformation mechanism(s) leading to inelastic compaction. Experiments on Slochteren sandstone were performed at constant strain rate, under conventional triaxial and zero-lateral strain (uniaxial or 1D vertical strain) boundary conditions at strain rates of 10^–6^ to 10^–8^ s^−1^, and at a constant stress. Systematic investigation of the effect of temperature (room temperature, 100 °C, 130 °C) and pore fluid pH (7 and 12.3) allowed for identification of the main deformation mechanism(s) operating in the compacting Slochteren sandstone, as stress corrosion cracking and pressure solution theories and data, together with quartz dissolution kinetics theory and data, suggest that those processes are dependent on temperature and fluid pH (e.g. Dove 1995; Knauss and Wolery 1988; Pijnenburg and Spiers 2020; Rimstidt and Barnes 1980). The mechanical data were complemented by microstructural investigation of undeformed and deformed samples, to further constrain the inelastic deformation mechanism(s) operating at the grain-scale. Ultimately, our results are used to make inferences about the amount of inelastic compaction that may still occur in the Groningen gas field now that production has stopped.

## Background on the Groningen gas field and Slochteren sandstone

The Groningen gas field, situated in the northeastern Netherlands, spans approximately 900 km^2^ and stands as the largest onshore gas reservoir in western Europe. Discovered in 1959, its initial gas in place (GIIP) was estimated to be around 2900 billion m^3^ (De Jager and Visser 2017). Gas extraction started in 1963 and as of 2023, nearly 80% of the gas has been extracted from the reservoir (NAM 2024), reducing the pore pressure from the initial 35 MPa to about 6–17 MPa (NAM 2019). The resulting increase in effective vertical stress caused compaction at depth, at the level of the heavily faulted reservoir. Though the region is tectonically inactive, the first seismic event was recorded in 1991 (Van Thienen-Visser and Breunese 2015). Since then, seismic events have increased in frequency and magnitude as gas production continued, culminating in the largest seismic event, which occurred at the village of Huizinge in 2012 with a moment magnitude of 3.6. Following the Huizinge earthquake, an increase in social and political pressure resulted in a stepwise reduction in production rate from 54 billion cubic meters per year (bcm/y) in 2013 to 20 bcm/y in 2018 (Vlek 2019), in an attempt to curb the induced seismicity. Eventually, the Dutch Government decided to completely abandon the field and production has stopped on April 19, 2024 (Ministry of Economic Affairs and Climate Policy 2024).

Evaluation of the location and magnitude of the induced earthquakes has shown that they are strongly correlated to the subsidence observed at the surface (Bourne et al. 2014). In-situ measurements suggest that the surface subsidence is largely caused by compaction at the reservoir level (Cannon and Kole 2017). The gas-bearing reservoir rock consists of the Slochteren sandstone, which is part of the extensive Upper Rotliegend Group. It is positioned at roughly 3 km depth, with a thickness varying from 100 to 250 m (De Jager and Visser 2017). It is composed primarily of aeolian, alluvial, and fan deposits (De Jager and Visser 2017), resting directly on top of Carboniferous shales that are the source rock for the gas. The Slochteren formation is overlain by the 50-m thick Ten Boer claystone, followed by a thick evaporite sequence (500–1000 m), the Zechstein formation (Amthor and Okkerman 1998).

The Slochteren Sandstone is composed of quartz (72–90 vol.%), feldspar (8–25 vol.%), clay (0.5–5.5 vol.%), and lithic fragments (3–10 vol.%), which include basaltic and sedimentary lithoclasts (Waldmann et al. 2014; Waldmann and Gaupp 2016). The mean size of the quartz and feldspar grains typically ranges between 150 and 250 $\upmu {\text{m}}$ (Pijnenburg et al. 2018), with thin clay films (1- to 10-$\upmu {\text{m}}$ thick) coating their surfaces and many grain contacts (Gaupp et al. 1993; Waldmann and Gaupp 2016; Pijnenburg et al. 2019b). Throughout the field, porosity varies from mean values of 12–16% at the fringes to 18–24% in the centre (NAM 2013).

## Materials and methods

We performed three types of deformation experiments on fluid-saturated Slochteren sandstone samples:

- Conventional triaxial compression experiments at constant strain rate, with ${\sigma }_{1}>{\sigma }_{2}={\sigma }_{3}={P}_{\mathrm{c}}$ (confining pressure), performed at *P*_c_ = 39 MPa and room temperature or 100 °C (abbreviated as “Triaxial tests”);
- Uniaxial strain triaxial compression experiments at constant strain rate, with ${\varepsilon }_{1}>{\varepsilon }_{2}={\varepsilon }_{3}=0$, performed at an initial *P*_c_ of 39 MPa and room temperature (referred to as “Uniaxial strain tests”); and
- A single constant stress (creep) experiment, conducted under conventional triaxial test conditions at *P*_c_ = 39 MPa and 130 °C (i.e. a “Creep test”).

The former two types of experiments were conducted to quantify the additional contribution of time- or rate-dependent deformation mechanisms to inelastic compaction, and to evaluate the effect of boundary conditions on these processes. The creep test was performed to evaluate the potential role of dissolution–precipitation, or pressure solution, in contributing to inelastic compaction.

All experiments conducted, along with their corresponding test conditions and key mechanical data, are summarised in Table 1.

**Table 1:** List of the experiments and key mechanical data obtained. In all experiments the pore pressure is 10 MPa. Note that in all experiments the confining pressure starts at 39 MPa and remains constant in the conventional triaxial compression and creep experiments, but is allowed to vary in the uniaxial strain experiments to maintain a zero-lateral strain boundary condition

Sample	TVD	*ϕ*	*T*	axial strain rate	pore fluid pH (initial value)	*Q*_peak_
	(m)	(%)	(°C)	(s^−1^)		(MPa)
**Conventional triaxial tests**						
SDM1-lo-6-tri	3060.3	14.5	20	1 × 10^–6^	7	121.8
SDM1-lo-8-tri	3060.3	15.2	20	1 × 10^–8^	7	103.2
ZRP3a-lo-6-tri	2940.2	16.1	20	1 × 10^–6^	7	93.0
ZRP3a-lo-8-tri	2940.2	16.2	20	1 × 10^–8^	7	90.0
SDM1-hi2-6-tri	2951.6	17.8	20	1 × 10^–6^	7	64.8
SDM1-hi2-8-tri	2951.6	17.8	20	1 × 10^–8^	7	62.7
SDM1-hi1-8-pH12-tri^*^	2933.5	17.5	20	1 × 10^–8^	12.3	n.a
SDM1-hi2-T100-6-tri^*^	2951.6	17.8	100	1 × 10^–6^	7	n.a
SDM1-hi2-T100-8-tri^*^	2951.6	18.2	100	1 × 10^–8^	7	n.a
SDM1-hi3-T100-8-pH7-tri^*^	2986.5	18.1	100	1 × 10^–8^	7	n.a
SDM1-hi3-T100-8-pH12-tri^*^	2986.5	18.9	100	1 × 10^–8^	12.3	n.a
**Uniaxial strain tests**						
SDM1-lo-6-uni	3060.3	14.6	20	1 × 10^–6^	7	n.a
SDM1-lo-8-uni	3060.3	14.9	20	1 × 10^–8^	7	n.a
SDM1-lo-8-pH12-uni	3060.3	14.9	20	1 × 10^–8^	12.3	n.a
ZRP3a-lo-6-uni	2940.2	16.1	20	1 × 10^–6^	7	n.a
ZRP3a-lo-8-uni	2940.2	16.0	20	1 × 10^–8^	7	n.a
SDM1-hi1-8-pH7-uni	2933.5	17.7	20	1 × 10^–8^	7	n.a
SDM1-hi1-8-pH12-uni^*^	2933.5	17.9	20	1 × 10^–8^	12.3	n.a
SDM1-hi2-6-uni	2951.6	18.5	20	1 × 10^–6^	7	n.a
SDM1-hi2-8-uni	2951.6	18.8	20	1 × 10^–8^	7	n.a
**Creep test**						
SDM1-hi3-creep	2986.5	17.8	130	1 × 10^–6^	7	n.a

TVD is the true vertical depth from which the sample was retrieved, $\phi$ is the starting porosity, $T$ is the temperature, ${Q}_{\mathrm{peak}}$ is the peak strength defined as maximum differential stress after which strain softening was observed. Silica-saturated solution was employed for the creep test

^*^samples used for microstructural analysis

### Materials and preparation

The Slochteren sandstone samples used in this study were obtained from core material retrieved by the field operator (Nederlandse Aardolie Maatschappij, NAM) from the Stedum (SDM)−1 well, drilled in 1965 prior to major gas production, and the Zeerijp (ZRP)−3a well, drilled in 2015. For the triaxial tests, a total of 10 cylindrical samples were cored of 17.5 mm in diameter and 34–36 mm in length, and 1 sample (SDM1-hi1-8-pH12-tri) of 24.7 mm in diameter and 51.4 mm in length, at different sampling depths. In addition, for the uniaxial strain and creep tests, a total of ten samples of 24.7 mm diameter and 50–52 mm length were retrieved from the same depths as the samples used for the triaxial tests, resulting in sample sets of 3–6 petrophysically nearly identical samples (so-called twin samples) for each sampling depth. All cores were obtained in an orientation perpendicular (within ~ ${10}^{\circ }$) to the often slightly inclined bedding. Their end faces were cut and ground square to ensure uniform load distribution. The porosity of each sample was measured using the water-saturation method, after which the sample was oven-dried at 50 $^\circ{\rm C}$ for a minimum of 2 weeks. The starting porosities of our samples were 14.5–15.2% (SDM1-lo series), 16.0–16.1% (ZRM3a-lo series), 17.5–17.9% (SDM1-hi1 series), 17.8–18.8% (SDM1-hi2 series) and 17.8–18.9% (SDM1-hi3 series), respectively (Table 1), which roughly covers the mean porosity values at the fringes and the centre of the gas field.

All experiments were performed using distilled water as the pore fluid, except for three experiments which employed a basic solution (SDM1-hi1-8-pH12-tri, SDM1-hi3-T100-8-pH12-tri and SDM1-hi1-8-pH12-uni) and one experiment which employed a silica-saturated solution (SDM1-hi3-creep). A high pH, sodium hydroxide (NaOH) solution (pH = 12.3 at $T=20^\circ{\rm C}$) and a silica-saturated solution (120ppm at $T=130^\circ{\rm C} , \text{pH}=7;$ Morey et al. 1962) were prepared by diluting a fixed quantity of pure ($>$ 99%) sodium hydroxide pellets and sodium silicate solution in distilled water, respectively. The solutions were thoroughly stirred and stored at room temperature in a sealed flask for at least seven days prior to use.

### Experimental methods

**Fig. 1:**
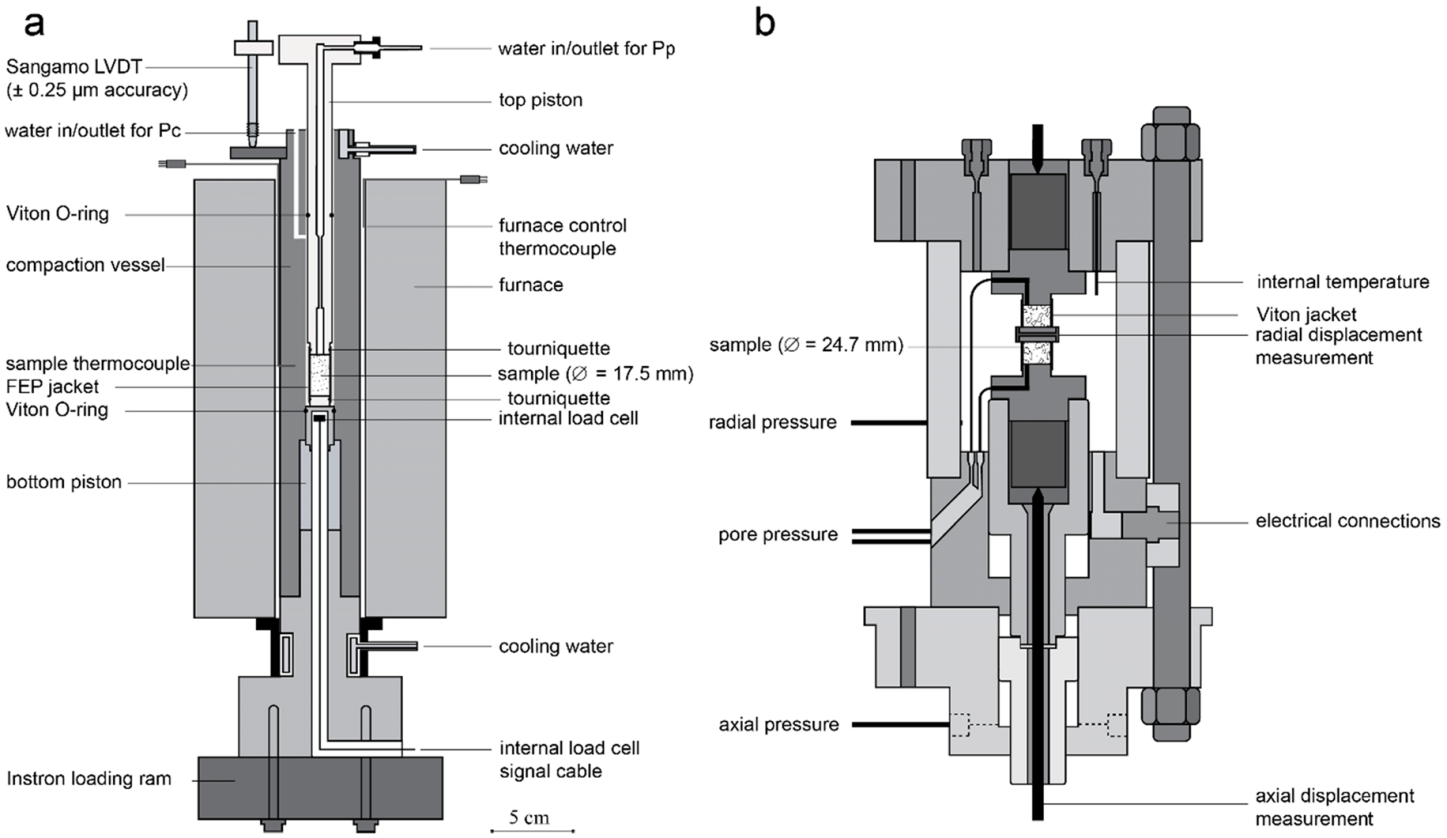
Schematic diagrams showing the internal details of **a** the deformation apparatus used for the constant strain rate experiments under conventional triaxial test conditions and **b** the deformation machine used for the experiments performed under uniaxial strain conditions and constant stress (creep) conditions (after Hol et al. 2018)

The conventional triaxial experiments (the “triaxial tests”) were performed using a small-scale triaxial deformation apparatus (Fig. 1a). The triaxial machine consists of an externally heated pressure vessel with water as the confining medium. The main pressure vessel contains the sample assembly (17.5 mm diameter). It can be pressurized up to 100 MPa confining pressure, with the pressure being kept nominally constant using a servo-controlled ISCO pump. Axial load is applied through an Instron-8862 servo-controlled loading frame at a constant displacement rate. The applied load is measured externally using the Instron load cell (0–100 kN, resolution ± 0.05 kN), allowing for applying an axial stress of up to ~ 415 MPa. Piston position and displacement is also measured using two linear variable differential transformers (LVDT), one located in the Instron drive unit (± 50 mm range, resolution ± 0.25 μm) and one located between the top piston and the pressure vessel (Sangamo; ± 1 mm range, resolution ± 0.1 μm), with the former used for piston position control, and the latter used for actual measurement. Another servo-controlled ISCO pump is used to apply a nominally constant pore pressure, and monitor any pore volume changes, for the duration of the experiment. Heating of the sample is done through a furnace (± 0.5 °C accuracy) and controlled using a K-type chromel–alumel control thermocouple, located within the furnace windings and connected to a proportional-integral-derivative (PID) controller. Sample temperature is measured independently through a second K-type thermocouple, embedded in the vessel wall adjacent to the sample.

The uniaxial strain tests and the single creep test were performed using a second, externally heated, triaxial deformation apparatus (Fig. 1b). This triaxial machine consists of a pressure vessel, containing the sample (24.7 mm diameter), with silicone oil (Shell Thermia B) as the confining medium. Servo-controlled Dustec Motor Driven Displacement Pumps (resolution ± 0.002 MPa) allow for up to 100 MPa confining pressure, as well as up to 100 MPa axial pressure, corresponding to ~ 97 MPa axial stress taking into account seal friction. Note that the latter limit prevents us from reaching failure under the confining conditions applied to the Slochteren sandstone in this study. Pore pressure can be applied up to pressures of 100 MPa using a similar Dustec pump, while any change in pore volume is recorded by the pore pressure pump. Displacements are measured in both the axial and radial directions. Axial displacement is measured using an LVDT (resolution ± 0.1 μm) located under the pressure vessel and connected to an externally located frame, transmitting the relative piston displacement to the sensor. Radial displacement is measured using a two-arm cantilever beam Wheatstone bridge (resolution ± 0.03 μm) in direct coupling with the outer surface of the sample via Viton-jacket punctures and steel pins located at the centre of the sample. Note that the sensitivity of the radial displacement bridge to elevated temperature limited its use to room temperature. Continuous feedback from the axial and radial displacement measurements is used to adjust the axial and confining pressures to maintain a nominally constant displacement rate and zero lateral displacement to achieve a uniaxial strain boundary condition, respectively.

### Testing procedures

For both experimental apparatuses, the fluid-saturated sample was covered by a sleeve (fluorinated ethylene propylene/FEP or Viton) and tightened against the upper and lower loading pistons. To reduce friction at the sample-piston interfaces, thin Teflon sheets (50 μm) were included at these locations during the jacketing procedure. For the uniaxial strain experiments, the radial displacement measurement system was now attached at the centre of the sample. After placing the assembly in the pressure vessel, the system was brought to a confining pressure (${P}_{\text{c}}$) of 15 MPa and a pore pressure (${P}_{\text{p}}$) of 10 MPa resulting in an effective confining pressure (${P}_{\text{eff}}={P}_{\text{c}}-{P}_{\text{p}}$) of 5 MPa. In case of an experiment at elevated temperature, the vessel was brought to the specified testing temperature. The system was subsequently left to equilibrate for 12–20 h, after which the confining pressure was increased to the target pressure of 39 MPa at a constant rate of ~ 0.1 (triaxial test) or ~ 0.01 MPa/s (uniaxial strain and creep tests), for the small-scale triaxial deformation apparatus (Fig. 1a) and for the second triaxial machine (Fig. 1b), respectively. The system was left to equilibrate for another two hours prior to starting the testing protocol for the different types of experiments.

#### Constant strain rate experiments under conventional triaxial test conditions (Triaxial tests)

To start the deformation experiment, the piston was advanced, using the servo-controlled loading frame, to apply axial load. The sample was axially deformed up to a total axial strain of ~ 1.5% at a prescribed constant strain rate of $1.0\times {10}^{-6}$ or $1.0\times {10}^{-8} \, {\mathrm{s}}^{-1}$, after which the piston was halted. Upon termination of each experiment, the sample was axially fully unloaded (${\sigma }_{1}-{\sigma }_{3}=0$ MPa) and the confining pressure was reduced to ${P}_{\mathrm{c}}=15$ MPa. Finally, the pore pressure was removed, followed by full removal of the confining pressure. Axial unloading and depressurization were achieved at a rate of ~ 0.01–0.1 MPa/s. If needed, the vessel was allowed to cool down to room temperature before carefully extracting the sample.

#### Constant strain rate experiments under uniaxial strain test conditions (Uniaxial strain tests)

Deformation was initiated by increasing the axial stress. The feedback between the axial LVDT and the axial pressure pump ensured that axial deformation occurred at a prescribed constant strain rate of $1.0\times {10}^{-6}$ or $1.0\times {10}^{-8} \, {\mathrm{s}}^{-1}$. Simultaneously, feedback between the radial displacement measurement and the radial pressure pump ensured the uniaxial strain (zero-lateral strain) condition was maintained. Once an axial stress of ~ 90 MPa (i.e. ~ 90% of the maximum axial stress that can be achieved using the apparatus; Fig. 1b) was reached, axial loading was halted, followed by axial unloading of the sample (${\sigma }_{1}-{\sigma }_{3}=0$ MPa) and a reduction of the confining pressure to $15$ MPa. Finally, the pore pressure was removed, followed by full removal of the confining pressure and careful extraction of the sample from the vessel. The depressurization rate employed was ~ 0.01 MPa/s.

#### Creep experiment under conventional triaxial test conditions (Creep test)

During sample assembly for the long-term creep experiment, a single crystal quartz plate (Alineason Materials Technology GmbH) with a hole near the edge ($d=1\text{ mm}$), ensuring connectivity between the pore fluid pump and the saturated sandstone sample, was placed between the sample and the bottom piston. The quartz plate was intended to capture evidence of dissolution–precipitation or pressure solution, by means of grain contact imprints onto the disc (cf. Schutjens et al. 2021). Therefore, we used a single-sided polished disc of 25.4 mm in diameter and 3.0 mm in thickness, with the crystallographic z-axis of quartz pointing perpendicular to the face of the disc (polished end placed against the sample). After emplacement of the disc, setting up of the experiment continued as described above. After imposing a confining pressure of 15 MPa and a pore fluid pressure of 10 MPa, the vessel was heated to 130 $^\circ{\rm C}$ over a period of 3 days. Subsequently, the confining pressure was increased to $39$ MPa, and deformation was induced at a prescribed constant strain rate of $1.0\times {10}^{-6} \, {\text{s}}^{-1}$ up to an axial stress of ~ 68 MPa, equivalent to vertical, maximum principal stress for the Groningen gas field (Van Eijs 2015). The applied stress was kept constant and the sample was allowed to creep for ~ 400 h, while maintaining the confining pressure and pore pressure constant. At the end of the experiment, the sample was fully unloaded (${\sigma }_{1}-{\sigma }_{3}=0$ MPa) and the confining pressure decreased to $15$ MPa. After removal of the pore pressure, the remaining confining pressure was removed and the vessel was allowed to cool down to room temperature before the sample was carefully extracted.

### Data acquisition and processing

In all the triaxial experiments, external axial load, confining pressure, pore pressure, pore fluid volume change, sample temperature, and Instron and Sangamo LVDT displacements were logged every 1 s during hydrostatic loading. During the axial loading stage, data was logged every 1 s in the experiments with an axial strain rate of $1.0\times {10}^{-6}$ s^−1^ and every 5 s in the slower experiments performed at $1.0\times {10}^{-8} {\text{s}}^{-1}$. Apparatus distortion was corrected using calibrations with a metal dummy of known elastic properties carried out under pressure–temperature conditions corresponding to the present testing conditions. The logged data were processed to yield the total axial strain ${\varepsilon }_{1}$, the differential stress ($Q={\sigma }_{1}-{\sigma }_{3}$), the total porosity reduction $\Delta \phi$ (i.e. change in sample pore volume divided by the initial sample volume), and the mean effective stress ($P={(\sigma }_{1}+2{\sigma }_{3})/3 - {P}_{\text{p}}$) versus time.

In the uniaxial strain and creep experiments, axial and confining pressure, pore pressure, pore fluid volume change, sample temperature, and axial and radial displacement signals were logged every 10 s during hydrostatic loading, every 10 or 30 s during the axial loading stage at $1.0\times {10}^{-6}$ or $1.0\times {10}^{-8} \, {\text{s}}^{-1}$, respectively, and every 30 s during the creep stage. Apparatus distortion and frictional forces resulting from the O-ring seals were corrected using pre-determined calibrations, similar as for the triaxial tests. These data were processed to yield ${\varepsilon }_{1}$, ${\sigma }_{1}$, $\Delta \phi$ and $P$ as a function of time.

### Microstructural analyses

Upon retrieval from the experimental apparatus, the samples were dried in an oven at 50 °C for at least 2 weeks. After drying, selected deformed and undeformed samples were impregnated with blue-coloured resin and sectioned parallel to the sample axis (Dettmar dissection Technology GmbH&Co.KG). The smaller samples were sectioned over their full sample width and length (section dimension of 17.5 by 34–36 mm), while thin sections of the larger samples covered the full sample width and approximately three quarters of the total sample length, including one end of the samples (section dimension of 24.7 by 38–40 mm). Each section was imaged in full using a ZEISS Gemini 450 scanning electron microscope (SEM) operated in backscatter electron (BSE) mode with a resolution of 0.80 $\upmu {\text{m}}$ (acceleration voltage: 15 kV; beam current: 1.0 nA; working distance: 10 mm). Automatic tile stitching was done using ZEISS Atlas 5 software. The undeformed samples were analysed further using electron dispersive X-ray spectrometry (EDX) mounted on ZEISS EVO 15 SEM, for sections of 6 to 9 ${\text{mm}}^{2}$ in total area (comprising 2 to 3 images of 3 ${\text{mm}}^{2}$ each per section), with a resolution of 1.0 $\upmu {\text{m}}$ (acceleration voltage: 15 kV; beam current: 1.0nA; working distance: 10 mm). Further processing of the images obtained for the undeformed samples was done to yield clay phase maps derived from measurements of the main constituent elements, i.e. Si, Al, K, Na, Ca, Mg, Fe and Ba, following the filtering method described in great detail in Shinohara et al. (2024). Table 2 lists the filtering sequence carried out to compile each phase map. These clay phase maps were processed further to construct intergranular clay phase maps by isolating the clays present within grain contacts (i.e. where quartz, feldspar, other lithic fragments are juxtaposed within ~ 10 $\upmu {\text{m}}$ proximity from each other). This excludes clays present in the pore space or coating the pore walls, as they do not contribute to the load-bearing framework.

**Table 2:** Formulas used to convert single element maps to phase maps

Mineral (group)	Elements in mineral phase	Formula
Quartz	Si, O	[Si]-[Al]-[K]-[Na]
K-feldspar	K, Al, Si, O	[K]-[Na]-quartz, particle size ≥ 314 μm^2^
Plagioclase	Na	[Na] + ([Ca] ∩ [Al])-quartz-K-feldspar-[Mg], particle size ≥ 314 μm^2^
Dolomite + siderite + barite	Ca, Mg, Fe, Ba	[Ca] + [Mg] + [Fe] + [Ba]-quartz-K-feldspar-plagioclase
Clay minerals	Al, Si, O	[Al]-quartz-K-feldspar-plagioclase-dolomite-siderite-barite

Crack density analysis was performed on seven samples (one undeformed and six deformed samples—see Table 1) along 3-mm wide axial profiles covering the full length of each thin section, using the stitched BSE micrographs. Individual visible cracks (i.e. intergranular alignment of black pixels) were traced manually over a total area of 102–120 ${\text{mm}}^{2}$, focusing only on the main framework grains (i.e. quartz, K-feldspar and plagioclase). This method has an acceptable and reproducible error of $\pm 15\%$ in crack number count for each map (Verberne et al. 2021). Sample (2D) porosity ${\phi }_{\text{im}}$ is determined from thresholding filtered (Gaussian blur, pixel radius 2) BSE images using the Otsu algorithm (Otsu 1979). Crack orientation distribution and total crack density ${\rho }_{\text{cr}}$ (${\mathrm{mm}}^{-2}$), defined as ${\rho }_{\text{cr}}={N}_{\text{cr}}/({A}_{\text{map}}(1-{\phi }_{\text{im}}))$, where ${N}_{\text{cr}}$ is the number of cracks and ${A}_{\text{map}}$ is the area of domain in consideration were obtained along the length of each sample. To remove the potential sample end effects, cracks mapped in the upper 5 mm of the thin section (i.e. top of the sample) were not considered for the crack orientation distribution. The surface of the quartz plate in contact with the sandstone sample during the creep experiment (SDM1-hi3-creep) was also analysed, before and after the deformation, using the ZEISS Gemini 450 SEM. The quartz surface was inspected for any features representative of mass transfer processes or pressure solution (e.g. pits with surface morphology of indenting grains and precipitation of the dissolved material; see Schutjens et al. 2021).

## Results

### Mechanical data

We adopt the convention that compressive stresses, compressive axial strains, compressive volumetric strains and porosity reduction (i.e. compaction) are defined as positive. In our experiments, the principal compressive stresses are denoted as ${\sigma }_{1}>{\sigma }_{2}={\sigma }_{3}={P}_{\text{c}}$, where ${P}_{\text{c}}$ is the confining pressure. We define peak strength (${Q}_{\text{peak}}$) as the maximum differential stress ${({\sigma }_{1}-{\sigma }_{3})}_{\text{max}}$ after which strain softening was observed.

#### Triaxial and uniaxial strain test data

**Fig. 2:**
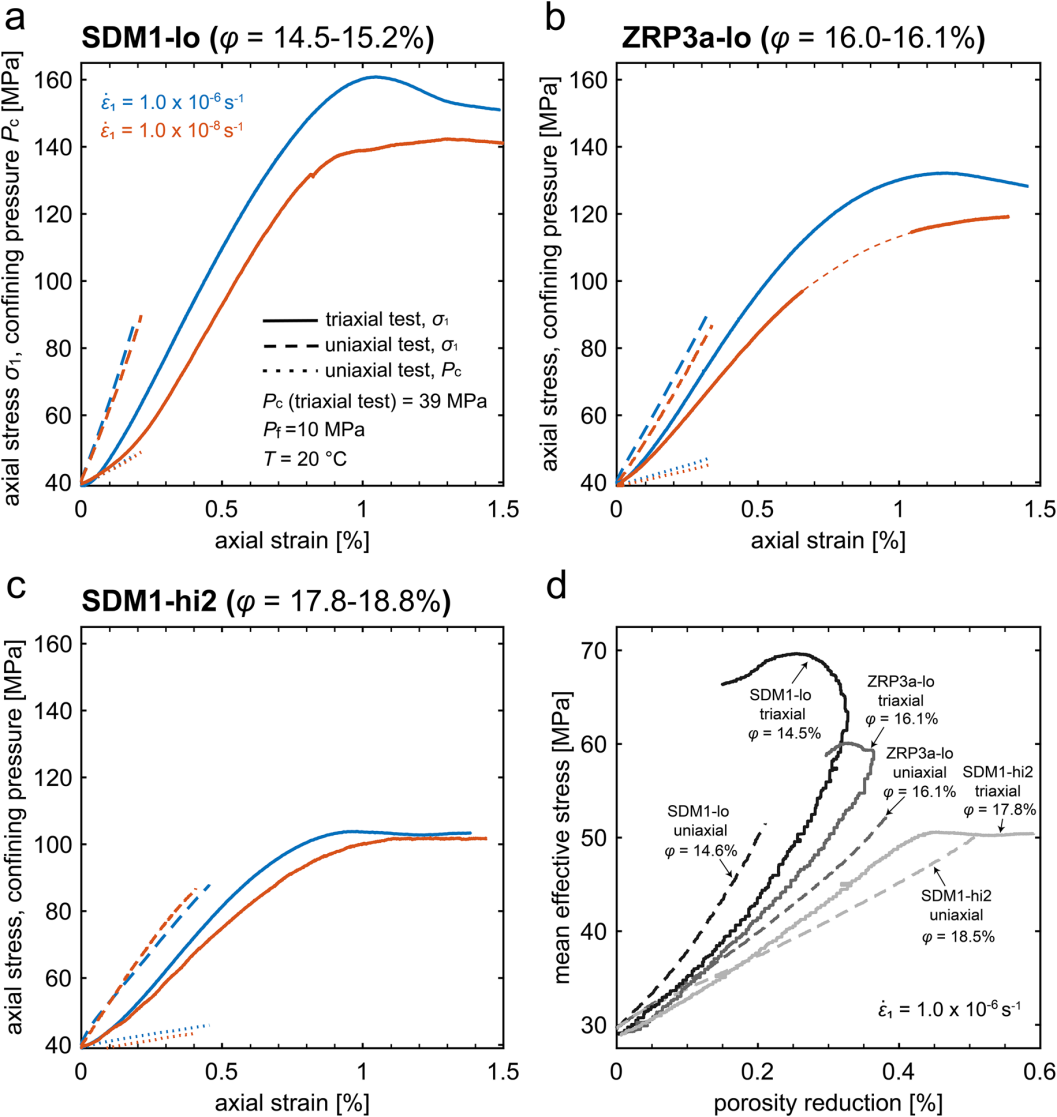
Plots showing the mechanical data obtained in our constant strain rate experiments on water-saturated Slochteren sandstone samples deformed at $20^\circ{\rm C}$ under conventional triaxial (solid lines) and uniaxial strain (dashed lines) conditions. Axial stress (${\sigma }_{1}$) and confining pressure (${P}_{\text{c}}$) versus axial strain (${\varepsilon }_{1}$) curves obtained for samples with a porosity of **a** $\phi =$ 14.5–15.2% (SDM-lo), **b** $\phi =$ 16.0–16.1% (ZRP3a-lo) and **c** $\phi =$ 17.8–18.8% (SDM1-hi2). The strain rate in each experiment was either $1.0\times {10}^{-6}$ (blue curves) or $1.0\times {10}^{-8} \, {\text{s}}^{-1}$ (red curves), as indicated. Note that in **b** part of the data is missing in the slow experiment ($1.0\times {10}^{-8} \, {\text{s}}^{-1}$), at axial strains from ~ 0.7 to 1.0% (dashed part of the red curve) due to a logging error. **d** Mean effective stress ($P$) versus porosity reduction ($\Delta \phi$) curves obtained for samples deformed at an axial strain rate ${\dot{\varepsilon }}_{1}$ of $1.0\times {10}^{-6} \, {\text{s}}^{-1}$. Note that axial loading during uniaxial strain experiments was halted at an axial stress of ~ 90 MPa, as it is ~ 90% of max. axial stress that can be achieved using the apparatus

Mechanical data, obtained during the constant strain rate experiments under conventional triaxial and uniaxial strain conditions performed on Slochteren sandstone samples with porosities in the range of 14.5 to 18.8%, deformed at room temperature, are shown in Fig. 2.

All samples deformed under conventional triaxial test boundary conditions showed a transition from initial, nonlinear, concave-up behaviour, to near-linear behaviour around axial stresses of 60–70 MPa (differential stresses of 21-31 MPa;solid lines, Fig. 2a–c). At faster loading rates (i.e. ${\dot{\varepsilon }}_{1}=1.0\times {10}^{-6} \, {\mathrm{s}}^{-1}$), peak stress was reached at 0.9–1.1% strain, followed by strain softening behaviour towards a steady residual stress attained at 1.2–1.5% strain. In the slower loading experiments (i.e. ${\dot{\varepsilon }}_{1}=1.0\times {10}^{-8} \, {\mathrm{s}}^{-1}$), no clear peak stress was observed and the little to no strain-softening occurred. Overall, peak stress, as well as the slope of the curves (i.e. apparent Young's modulus), was lowered with increasing porosity (cf. Figure 2a, 2b vs. 2c), and decreasing axial strain rate during the entire loading phase (cf. blue and red lines in Fig. 2).

By contrast, samples deformed under uniaxial strain boundary conditions showed that the stress–strain behaviour was negligibly dependent on the imposed strain rate (dashed lines, Fig. 2a-c). Overall, all samples showed near-linear behaviour up to the maximum imposed axial stress of 90 MPa. Furthermore, during compression, confining pressure increased monotonically to ensure zero-lateral strain conditions, in all samples (i.e. to stop the samples from expanding radially). Though differences are small, it appears that at slower deformation rates, the change in confining pressure required to maintain a uniaxial strain boundary conditions is less, with the amount of difference seems to increase with increasing porosity (i.e. the total ${P}_{\text{c}}$-increase over the entire experiment, for all porosities, is about 1–2 MPa lower at a strain rate of 10^–8^ s^−1^ than at a strain rate of 10^–6^ s^−1^; see Fig. 2a–c).

Mean stress vs. porosity reduction data is shown in Fig. 2d for the faster loading experiments (i.e. ${\dot{\varepsilon }}_{1}=1.0\times {10}^{-6} \, {\text{s}}^{-1}$). Note that, for the slower loading experiments (i.e. ${\dot{\varepsilon }}_{1}=1.0\times {10}^{-8} \, {\text{s}}^{-1}$), volume data is not available since the magnitude of volume change due to pore fluid leakage and temperature variations is of the same order of magnitude as the sample pore volume change. For all samples deformed under conventional triaxial and uniaxial strain conditions, initial compactive behaviour was observed, with a clear lowering of the slope of the *P*–Δ*ϕ* curves with increasing porosity (i.e. decreasing apparent bulk modulus, Fig. 2d). In the triaxial experiments on lower porosity samples (i.e. SDM1-lo with $\phi =14.5\%$, and ZRP3a-lo with $\phi =16.1\%$; solid lines in Fig. 2d), volume data showed non-linear concave-up mean effective stress vs. porosity reduction behaviour, followed by an upward turn leading to net dilation. This dilation (i.e. porosity increase) corresponds to the strain softening behaviour observed in the differential stress–axial strain curves (Fig. 2a, b). By contrast, in the triaxial experiment on a higher porosity sample (SDM1-hi2 with $\phi =17.8\%$), quasi-linear stress vs. porosity reduction behaviour was observed up to $P=50$ MPa, followed by deviation from linearity characterized by compaction, again corresponding to the strain softening behaviour observed in the differential stress–axial strain curve (Fig. 2c). On the other hand, for all samples deformed in the uniaxial strain experiments (dashed lines in Fig. 2d), quasi-linear mean effective stress vs. porosity reduction behaviour was observed up to a mean effective stress of 50–55 MPa.

#### Effect of pore fluid pH and temperature on deformation behaviour (triaxial and uniaxial strain tests)

**Fig. 3:**
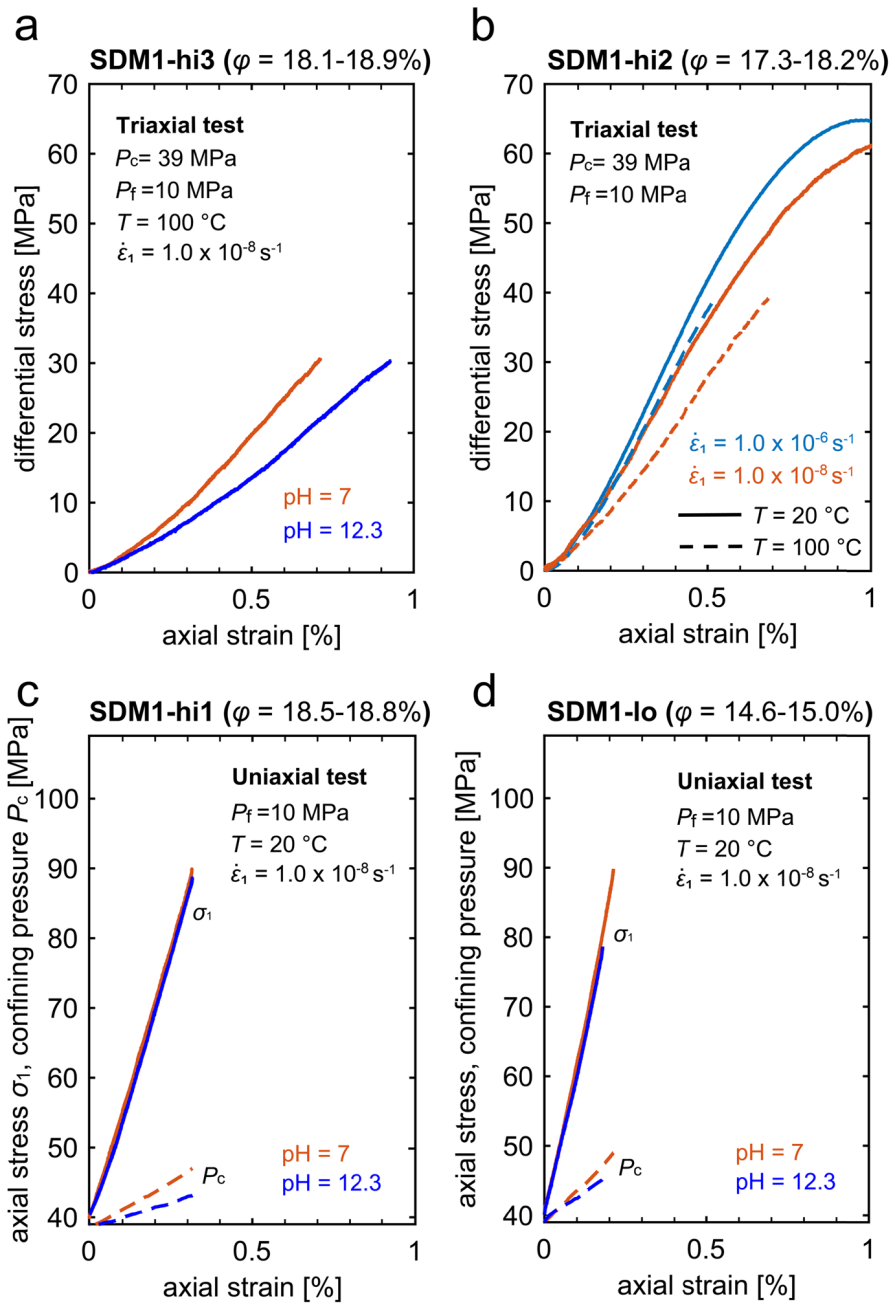
Stress–strain curves for constant strain rate experiments performed on Slochteren sandstone saturated with pore fluid of varying pH and deformed at varying temperature under conventional triaxial and uniaxial strain conditions. **a** Differential stress ($Q$) vs. axial strain (${\varepsilon }_{1}$) data as a function of pore fluid pH (pH 7, red curves; pH12.3, blue curves) under conventional triaxial condition (${P}_{\text{eff}}=29 \text{MPa}$, $T=100^\circ{\rm C}$). **b** $Q$ vs. ${\varepsilon }_{1}$ data obtained on highly porous samples (SDM-hi2, $\phi =$ 17.3–18.2%) deformed under conventional triaxial conditions (${P}_{\text{c}}$ = 39 MPa) at room temperature (solid lines) or 100 $^\circ{\rm C}$ (dashed lines), at either a fast (blue curves) or slow axial strain rate (red curves). Axial stress (${\sigma }_{1}$) and confining pressure (${P}_{\mathrm{c}}$) data vs. ${\varepsilon }_{1}$ obtained under uniaxial strain conditions (initial ${P}_{\text{c}}$ = 39 MPa, room temperature) for samples with **c** high porosity (SDM1-hi1, $\phi =$ 18.5–18.8%), and **d** low porosity (SDM1-lo, $\phi =$ 14.6–15.0%)

Stress–strain data for triaxial and uniaxial strain experiments systematically investigating the impact of pore fluid pH and elevated temperature are presented in Fig. 3. In general, the experiments showed qualitatively similar behaviour as the experiments presented in Fig. 2a–c, using distilled water and performed at room temperature. In the triaxial experiments on high porosity Slochteren sandstone, a lowering of the stress–strain curve was observed with increasing pore fluid pH from 7.0 to 12.3, suggesting a weakening of the material when saturated with a high-pH fluid (Fig. 3a). By contrast, in the uniaxial strain experiments pore fluid pH does not appear to affect the axial stress vs. axial strain behaviour (Fig. 3c, d). However, the pH effect was evident in the evolution of the confining pressure, with the rate of increase in confining pressure being smaller in the experiment with the higher pore fluid pH (i.e. the high-pH sample required a lower confining pressure to stop it from radially expanding than the neutral-pH sample). Though data is limited to four experiments, for a higher porosity sample, the pH-effect on the *P*_c_-change is enhanced (Fig. 3c vs. d). In addition, there appears to be a rough increase in *P*_c_-increase required to maintain zero-lateral strain boundary conditions with decreasing porosity.

At elevated temperature ($T=100^\circ{\rm C}$), experiments performed under conventional triaxial conditions showed similar stress–strain behaviour as at $T=20^\circ{\rm C}$, meaning a lowering of the stress–strain curve was observed with decreasing axial strain rate (Fig. 3b). At the same strain rate, sample softening was observed when increasing temperature from $T=20$ to $100^\circ{\rm C}$ (cf. solid vs dashed curves of the same colour). The magnitude of the softening resulting from increasing temperature is equivalent to that of about a two orders of magnitude decrease in strain rate (i.e. the stress–strain curve obtained at a strain rate of $1.0\times {10}^{-8} \, {\mathrm{s}}^{-1}$ at $T=20^\circ{\rm C}$ is similar to that obtained at $1.0\times {10}^{-6} \, {\mathrm{s}}^{-1}$ at $T=100^\circ{\rm C}$; cf. red solid curve vs. blue dashed curve).

#### Creep test data

**Fig. 4:**
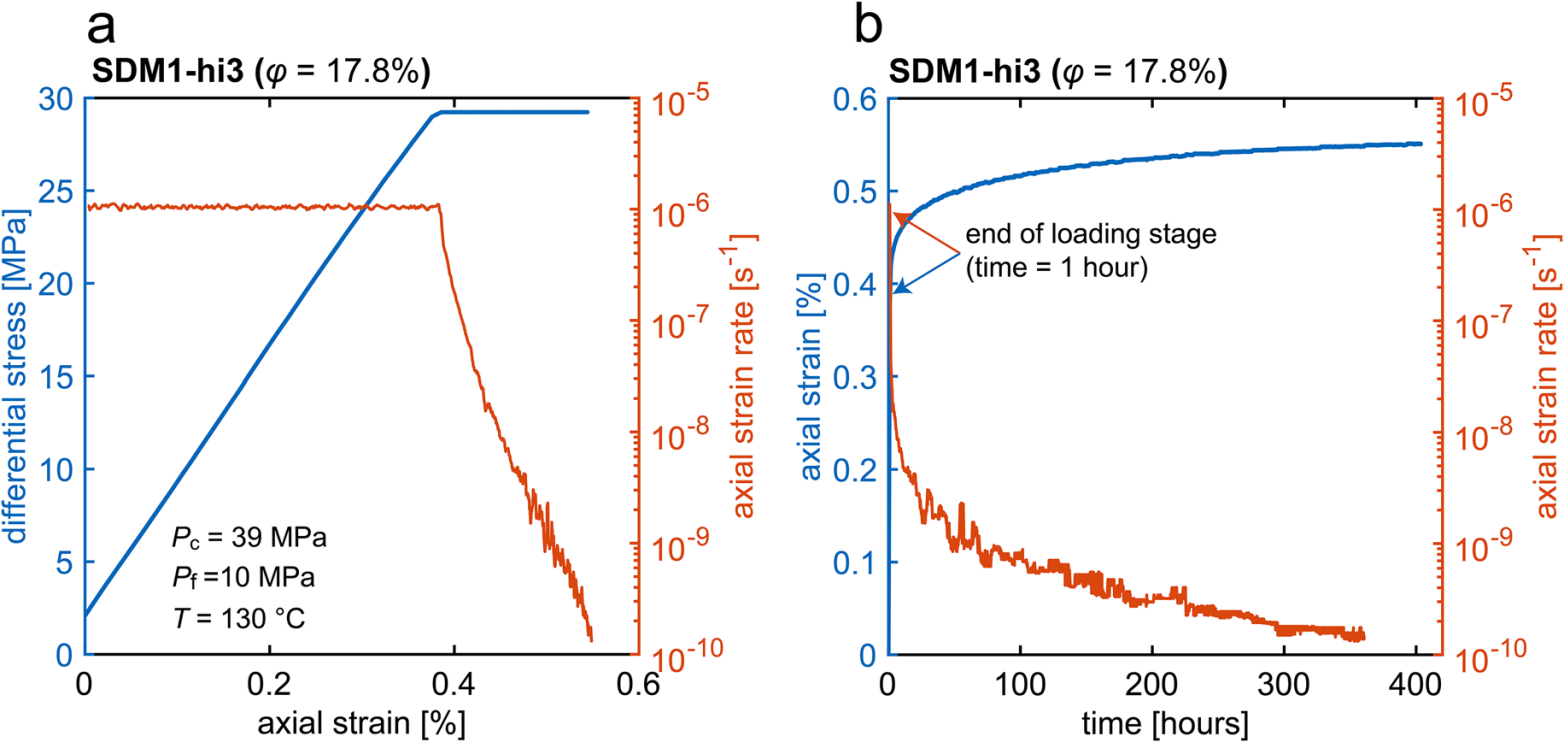
Mechanical data obtained in our creep experiment performed on a high porosity sample (SDM1-hi3; $\phi =17.8\%$) at $T=130^\circ{\rm C}$ under conventional triaxial test conditions. The creep experiment was conducted at a constant differential stress of ~ 29 MPa following initial axial loading at a constant strain rate of $1.0\times {10}^{-6} \, {\mathrm{s}}^{-1}$. **a** Differential stress and axial strain rate vs. axial strain. **b** Axial strain and axial creep strain rate vs. time

The differential stress and axial strain rate vs. axial strain, and the axial strain and axial strain rate vs. time data for the creep experiment performed at elevated temperature ($T=130^\circ{\rm C}$, duration = 17 days) are shown in Fig. 4. During the loading stage (constant strain rate of $1.0\times {10}^{-6} \, {\mathrm{s}}^{-1}$), near-linear behaviour was observed up to the start of the constant stress (i.e. creep) phase at a differential stress of 29 MPa (Fig. 4a). During the creep phase, the axial strain rate decreased monotonically, initially rapidly, during the first couple of hours, down to a strain rate of ~${10}^{-8} \, {\text{s}}^{-1}$ followed by a slower decrease down to a strain rate of ~${10}^{-10} \, {\text{s}}^{-1}$ after ~ 400 h of creep (Fig. 4b). Overall, permanent strains of 0.06% and 0.10% were added while the creep strain rate decreased from ${10}^{-6}$ to ${10}^{-8} \, {\text{s}}^{-1}$ and from ${10}^{-8}$ to ${10}^{-10} \, {\text{s}}^{-1}$, respectively (Fig. 4a).

#### Microstructural analysis

**Fig. 5:**
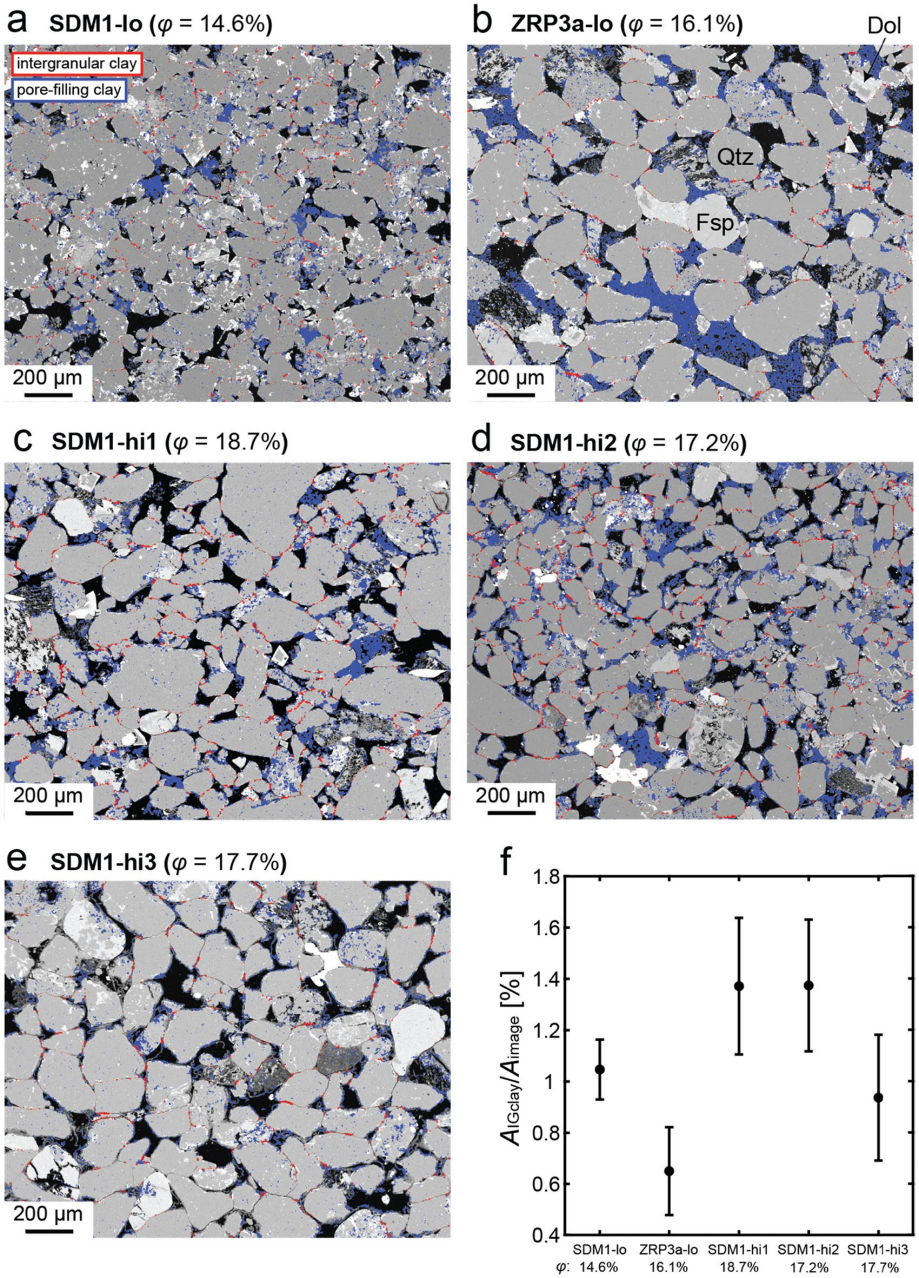
Representative clay maps superimposed onto back scattered electron (BSE) images of thin sections of undeformed starting material: **a** SDM1-lo, **b** ZRP3a-lo, **c** SDM1-hi1, **d** SDM1-hi2 and **e** SDM1-hi3. Overall, the Slochteren sandstone consists of quartz (Qtz), feldspar (Fsp), clay minerals (illite and kaolinite—not distinguished between in the clay maps) and dolomite (Dol) cement. Intergranular clays are highlighted in red, while pore-filling/coating clays are highlighted in blue. **f** The total intergranular clay fraction for each image starting material. Error bars show standard deviation, as obtained from intergranular clay fraction data measured in images taken at various locations within each thin section

Representative SEM–EDX micrographs of undeformed Slochteren sandstone samples obtained from each sampling depth are presented in Fig. 5a–e. In general, all samples consist of sub-angular to sub-rounded quartz with lesser amounts of feldspar. Clay minerals are extensively observed as pore-filling clay (shown in blue), clay films coating pore walls (shown in blue) and within grain contacts (intergranular clay; shown in red). All samples have comparable intergranular clay contents, ranging from 0.5 to 1.6% (Fig. 5f—see also Verberne et al. 2021).

**Fig. 6:**
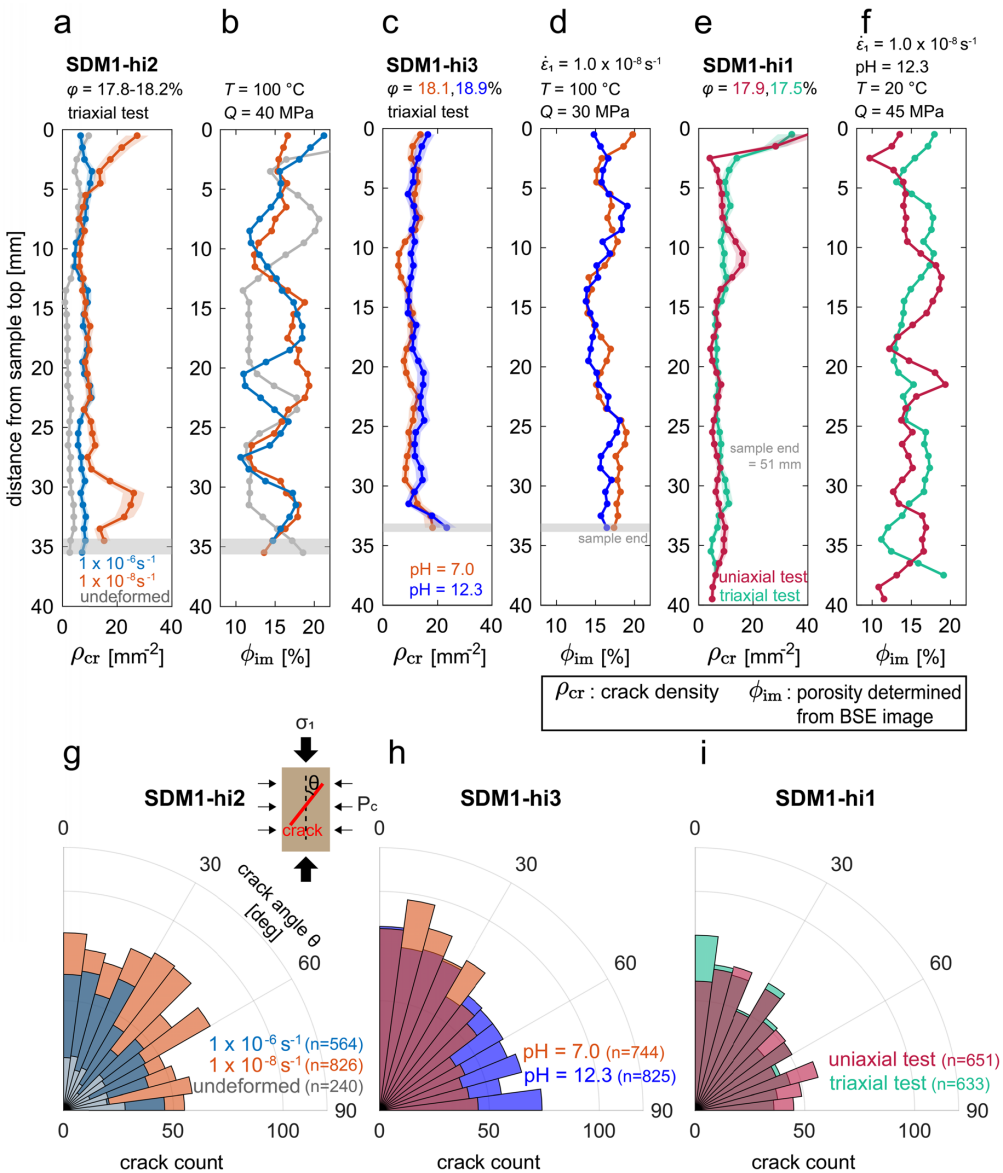
Plots showing intragranular crack density and porosity distributions as a function of distance from the sample top for **a**–**b** an undeformed SDM-hi2 starting material (grey) and samples deformed to a differential stress $Q$ of 40 MPa at $T=100^\circ{\rm C}$ and ${\dot{\varepsilon }}_{1}=1.0\times {10}^{-6}$ (blue) and $1.0\times {10}^{-8} \, {\mathrm{s}}^{-1}$ (orange) under triaxial conditions, **c**–**d** SDM1-hi3 samples deformed to a differential stress of 30 MPa at $T=100^\circ{\rm C}$, ${\dot{\varepsilon }}_{1}=1.0\times {10}^{-8} \, {\mathrm{s}}^{-1}$ and pore fluid pH of 7 (orange) and 12.3 (purple) under triaxial conditions, and **e–f** SDM1-hi1 samples deformed to a differential stress of 45 MPa under triaxial (green) and uniaxial strain (red) condition at $T=20^\circ{\rm C}$ and axial strain rate (${\dot{\varepsilon }}_{1}$) of $1.0\times {10}^{-8} \, {\mathrm{s}}^{-1}$. Crack orientation distribution for the **g** SDM1-hi2, **h** SDM1-hi3 and **i** SDM1-hi1 samples. The cracks were traced onto stitched, backscattered electron images of undeformed material and deformed samples

Crack density (${\rho }_{\mathrm{cr}}$), crack orientation distribution and 2D porosity (${\phi }_{\mathrm{im}}$) variation along the sample axis are presented in Fig. 6, for both selected undeformed and deformed Slochteren sandstone samples. Overall, deformed samples show a higher crack density than their undeformed counterpart (Fig. 6a). For samples deformed at a slow strain rate (10^–8^ s^−1^, Fig. 6a, c, e), the crack densities close to at least one end of the sample are 60–400% higher than the average crack density observed for the rest of the sample. This may be related to end effects, which appear to be enhanced when loading is slow, as the crack density of the sample deformed at a faster strain rate (10^–6^ s^−1^, Fig. 6a) negligibly increased at both ends of the sample. However, it could also be related to sample variability and/or local porosity variations, where local high porosity zones could lead to stress concentrations, which combined with stress corrosion cracking, could lead to more grain failure in slower experiments (cf. bottom end of fast and slow deformed samples SDM-hi2, Fig. 6a). More data is needed to conclude what caused enhanced cracking close to sample ends, though it should be noted that for the more central parts of the samples (5–25 mm from the top) crack densities of the deformed samples are very similar. Crack orientation distribution patterns of the triaxially deformed, water-saturated SDM1-hi2 samples showed negligible difference among the three samples (Fig. 6g).

Crack densities in samples deformed under triaxial boundary conditions employing pore fluid with a varying pH showed an increase in ${\rho }_{\mathrm{cr}}$ of ~ 10% when increasing pH from 7.0 to 12.3 (Fig. 6c, h). Furthermore, in the presence of a high-pH fluid, crack orientation became more horizontal (i.e. near-perpendicular to the axial loading direction; Fig. 6h). The boundary conditions did not appear to impact the average crack density significantly (Fig. 6e, triaxial vs. uniaxial strain). However, under uniaxial strain (zero-lateral strain) conditions, more near-horizontal (i.e. near-perpendicular to the axial loading direction) cracks appear to form than when the sample is deformed under triaxial boundary conditions.

A two-sided Kolmogorov–Smirnov test (Smirnov 1939) and a two-sided Mann–Whitney U test (Mann and Whitney 1947) were conducted to assess the statistical significance of the difference in crack orientation distributions observed between experiments performed at different boundary conditions and pore fluid pH. The Kolmogorov–Smirnov test is typically used to determine whether two distributions statistically differ. By contrast, the Mann–Whitney U test typically is used to test whether two sample means are equivalent or not. In this study, a significance level of 0.05 was taken for both tests. The Kolmogorov–Smirnov test showed the probability that the two crack density populations are equivalent is 0.139 and < 0.001 for the samples deformed under different boundary conditions and with different pore fluid pH, respectively. On the other hand, the Mann–Whitney U test showed that the probability that the two means are equivalent is 0.0274 and < 0.001 for the boundary condition and pore fluid pH effect, respectively. For the effect of boundary condition, while the null hypotheses must be overthrown for a two-sided Mann–Whitney U test (i.e. probability < significance level), the null hypotheses cannot be overthrown for a two-sided Kolmogorov–Smirnov test (i.e. probability > significance level). This means that we cannot conclude that the crack orientation distributions are statistically different between conventional triaxial and uniaxial strain boundary conditions, despite the (subtle) difference observed by visual inspection. Concerning the effect of pore fluid pH, the tests imply that both null hypotheses can be overthrown, meaning that the two crack orientation distributions are indeed statistically different.

**Fig. 7:**
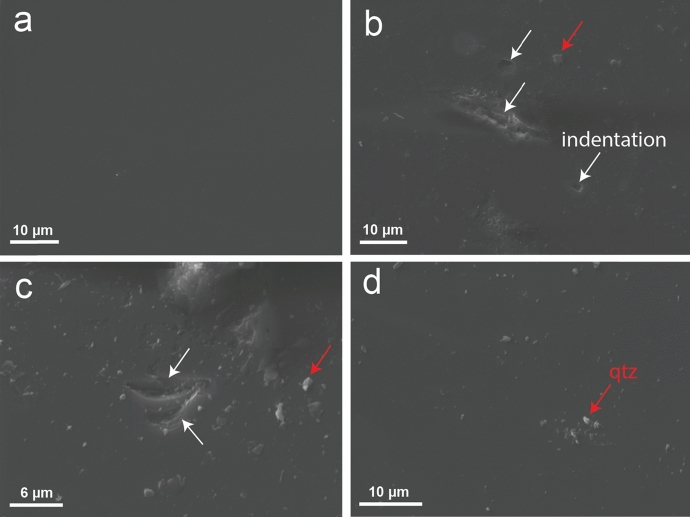
Secondary electron images of the surface of the quartz plate. **a** Surface of the quartz plate prior to the experiment. **b–d** Surface of the quartz plate after contact with a sandstone sample at a constant stress for 400 h at $T=130^\circ{\rm C}$, ${P}_{\text{c}}=39$ MPa and ${P}_{\text{f}}=10$ MPa. White arrows indicate features of grain indentation, with their surface morphology characterized by sharp ledges. Red arrows indicate small amounts of quartz precipitation on the surface of the quartz plate

Representative secondary electron micrographs of the surface of the quartz plate placed in the creep experiment are shown in Fig. 7, before and after the experiment. The surface of the undeformed quartz plate was smooth with no discernible features (Fig. 7a). By contrast, after being in contact with the Slochteren sandstone under stress and temperature, surface features include some grain indentations of 1–10 $\upmu {\text{m}}$ in size, with a surface morphology characterized by sharp ledges (Fig. 7b, c). Precipitation of small amounts of quartz is identified on the plate as well (Fig. 7b–d). Overall, little evidence for mass transfer related mechanisms was observed.

## Discussion

The constant strain rate experiments under conventional triaxial boundary conditions (triaxial experiments; at ${P}_{\text{eff}}$ = 29 MPa; $T=20^\circ{\rm C}$) showed a decrease in strength with decreasing strain rate (${\dot{\varepsilon }}_{1}$ from $1.0\times {10}^{-6}$ to $1.0\times {10}^{-8} \, {\mathrm{s}}^{-1}$; solid curves in Fig. 2a–c). The influence of pore fluid pH and temperature on the mechanical behaviour suggested a grain-scale deformation mechanism(s) that is enhanced by increasing $T$ and increasing pore fluid pH. Microstructural analyses of the deformed samples showed that fast deformation (strain rate of 10^–6^ s^−1^) resulted in an increase in crack density (Fig. 6a), which was further increased by deforming the material slower (strain rate of 10^–8^ s^−1^) or in the presence of a high pH pore fluid. By contrast, a negligible effect of loading rate on the axial stress vs. strain behaviour was observed in the experiments performed under uniaxial strain (zero-lateral strain) boundary conditions (uniaxial strain experiments; dashed curves in Fig. 2a–c), although the rate effect appears to be observed as the lowered increase in the confining pressure needed to maintain a uniaxial strain boundary conditions, with decreasing rate. Furthermore, the boundary condition of the experiment (i.e. triaxial vs. uniaxial tests) has negligible effect on crack density while the difference in crack orientation distributions is ambiguous (Fig. 6e, f, i). In the following, we will first discuss the microphysical mechanisms controlling the observed rate- or time-dependent deformation of the Slochteren sandstone. Then, implications for surface subsidence and induced seismicity in the Groningen gas field are discussed.

### Rate- or time-dependent deformation mechanisms in triaxial tests

For the stress-temperature-chemical conditions and strain rates employed in our experiments, the observed weakening could have involved 1) intergranular pressure solution (Rutter 1983), 2) slow, time-dependent crack growth (stress corrosion cracking—Atkinson 1984; Brantut et al. 2013, 2014; Heap et al. 2009a, b; Heap et al. 2009a, b; Heap et al. 2015; Pijnenburg et al. 2018; Shinohara et al. 2024) and/or 3) rate-dependent intergranular sliding (Bernabé and Brace 1990; Menéndez et al. 1996; Pijnenburg et al. 2018; Shinohara et al. 2024). We will discuss which one, or combination, of these three mechanisms can explain the rate-dependent deformation observed in the Slochteren sandstone. In case of intergranular sliding, this mechanism could lead to grain rearrangement in series with intragranular stress corrosion cracking, i.e. serial intergranular sliding is required for intragranular stress corrosion cracking to contribute to strain increments. On the other hand, intergranular sliding can also contribute to inelastic strain as an independent parallel process. To differentiate between the two modes of strain-contribution by intergranular sliding, we use the terms serial and parallel, respectively.

#### Intergranular pressure solution

In the presence of aqueous solutions, stressed granular materials may exhibit so-called intergranular pressure solution (IPS), where dissolution of grain material occurs at grain contacts under high normal stress, followed by diffusion through the grain boundary and precipitation of this material at lower stressed grain boundaries or pore walls (Spiers et al. 2004). In previous experiments where polished quartz surfaces are pressed against matrix grains, microstructures on the polished surface often show subrounded pits, deepening towards the pit-centre. These rough-surfaced pits reflect the surface morphology of indenting grains, indicating dissolution and are typically surrounded by precipitation microstructures, indicating overgrowths formed by the dissolved material (for example, see Fig. 6 in Schutjens et al. 2021). By contrast, on the surface of the quartz plate present in our creep experiment, we observed only irregular grain indentations characterized by sharp ledges (Fig. 7b, c), which are notably different from typical pressure solution features. Most likely this surface morphology feature was not caused by pressure solution, but rather by brittle failure and/or tensile fracturing due to the similarity in surface morphologies (e.g. Norton and Atkinson 1981; Schutjens et al. 2021). Furthermore, axial strain rates resulting from pressure solution are predicted to be on the order of ${10}^{-14}$ to ${10}^{-11} {\text{s}}^{-1}$ under our experimental (triaxial) conditions (${P}_{\text{eff}}=$ 29 MPa and $T=$ 20–130 $^\circ{\rm C}$), for sandstone with a grain size of 200 $\upmu {\text{m}}$ and porosity of $19\%$ (see Shinohara et al. (2024) for a detailed description of this calculation). These predicted creep rates are slower than the strain rates imposed or reached in any of the triaxial experiments (i.e. slowest strain rate of 10^–10^ s^−1^ was achieved in the creep experiment). Therefore, we infer that, it is unlikely that pressure solution played a significant role in controlling the time-dependent deformation observed in our triaxial experiments.

#### Stress corrosion cracking

Linear elastic fracture mechanics theory (Lawn 1993) describes how fractures propagate from flaws when the stress intensity factor of a material attains a critical level equal to the fracture toughness. In the presence of chemically active, aqueous fluids, chemical interactions between the fluid and the bonds at the stressed crack tip can lead to crack propagation at tensile stresses below those predicted by the stress intensity factor (i.e., stress corrosion; Anderson and Grew 1977; Atkinson 1984; Lawn 1993). Studies have shown that at high differential stress ($Q$ higher than the onset of inelastic dilatancy typically denoted as $C^{\prime}$—see e.g. Wong et al. 1997), time-dependent deformation of sandstones is indeed controlled by subcritical crack growth by stress corrosion (Brantut et al. 2013; Heap et al. 2009a, b; Heap et al. 2015; Ngwenya et al. 2001). Since (subcritical) crack growth is driven by the tensile stress at the tip of the crack, at a given stress state, the rate of sandstone deformation due to stress corrosion cracking increases with increasing porosity (e.g. Brantut et al. 2013), as contact stresses, and therefore the stresses that may act on surface flaws, are higher in more porous materials (Shinohara et al. 2025). This is in line with our data that the samples are weaker with increasing porosity, at given strain rates (Fig. 2a–c).

For our higher porosity samples (17.3–18.2%; SDM1-hi2 series), our microstructural analysis shows that the crack density increased locally (29–34 mm from the top of the sample, Fig. 6a) with decreasing strain rate from $1.0\times {10}^{-6}$ to $1.0\times {10}^{-8} \, {\mathrm{s}}^{-1}$ (Fig. 6a). This is consistent with subcritical crack growth being the potential grain-scale deformation mechanism for higher porosity samples, as cracks get more time to grow and lead to grain failure at slower loading rates.

Systematic investigation of the effect of temperature and fluid pH on the deformation behaviour of Slochteren sandstone with higher porosities (17.3–18.2%, SDM1-hi2 series; 18.1–18.9%, SDM1-hi3 series) showed weakening with increasing temperature and increasing pore fluid pH (Fig. 3a, b). In addition, when pH increases from 7 to 12.3, the crack density increased by ~ 10% (Fig. 6h). These mechanical data and microstructural observations are in line with experimental observation and theoretical considerations on stress corrosion cracking and crack growth velocity (Atkinson 1984; Atkinson and Meredith 1981; Darot and Guéguen 1986; Dove 1995), though we cannot completely rule out the possibility that the ~ 10% increase in crack density results from error in crack number counts (Verberne et al. 2021).

In addition, crack velocity is estimated to increase by a factor of 1929 and 338 when $T$ increases from 20 to 100 $^\circ{\rm C}$ and pH increases from 7.0 to 12.3, respectively (see Shinohara et al. 2024 for a detailed description of this calculation). This suggests more cracks are expected in an environment with pH 12.3 than with pH 7, which is in line with our microstructural observations for samples SDM-hi3-T100-8-pH7-tri and SDM-hi3-T100-8-pH12-tri (Fig. 6h). It is commonly assumed that the deformation rate is proportional to the crack velocity as $\dot{\varepsilon }\propto v$ (Brantut et al. 2014; Shinohara et al. 2024). If this holds, then, the predicted changes in crack velocity (3 orders of magnitude increase, when $T$ increases from 20 to 100 $^\circ{\rm C}$) would lead to proportional changes in the observed (equivalent) deformation rate. The similarity between the stress–strain curves obtained at $T=20^\circ{\rm C}$ and rate of ${10}^{-8} {\text{s}}^{-1}$, and $T=100^\circ{\rm C}$ and rate of ${10}^{-6} {\text{s}}^{-1}$ (see Fig. 3d), show that increasing temperature from 20 to 100 $^\circ{\rm C}$ led to increased additional strains equivalent to a decrease in strain rate by two orders of magnitudes. It is about 1 order of magnitude smaller than theoretical prediction though it should be kept in mind that the values of the input parameters have some uncertainty. In addition, the sample weakening observed with increasing pH (Fig. 3a) is at least qualitatively consistent with the crack velocity prediction. This correspondence between our mechanical observations and crack velocity theory suggests that SCC played a role in controlling deformation in our triaxial experiments on high-porosity Slochteren samples.

It should be noted that the subcritical crack growth in feldspar is not included in the above theoretical analysis. The Al–O bonds in aluminosilicates like feldspars make such an analysis more complicated than in quartz. However, previous experimental work has shown that cracking in feldspar sand is enhanced with increasing pore fluid pH from 5.5 to 11 (Hangx et al. 2010). Given the relatively low feldspar amount (8–25 vol.%) in Slochteren sandstone, and its dispersed spatial occurrence, framework support for the feldspar grains will be more effective and we expect that feldspar grain failure by SCC contributed less substantially to the time-dependent strain. Instead, the feldspar grains may continue to serve as passive markers, evidencing the small strain deformation that occurred, similar to the natural material (Verberne et al. 2021).

#### Parallel intergranular sliding

Under conventional triaxial boundary conditions, where the deforming material is allowed to compact or dilate in the radial direction, subcritical crack growth may be accompanied by serial intergranular sliding to accommodate the resulting deformation. However, if intergranular sliding occurs in our triaxial experiments, it may also be as a parallel process, i.e. independent of crack growth. It has been demonstrated that rate-dependent frictional sliding along grain contacts, similar to that seen in gouge material (Hunfeld et al. 2017), is possible in porous sandstones (De Waal and Smits 1988). Furthermore, the presence of low-cohesion, low-friction intergranular clay films may facilitate slip, further aiding dilation at higher axial stresses (${\sigma }_{1}-{P}_{\mathrm{c}}>{C}^{\prime}$; onset of inelastic dilatancy—see e.g. Wong et al. 1997). Considering the abundance of intergranular clay films in the Slochteren sandstone (Fig. 5a–e), it is therefore possible that a portion of the observed rate-dependence in our triaxial experiments (Fig. 2a–c) is a result of intergranular sliding facilitated by those thin clay films.

In samples with a relatively low porosity (i.e. <  ~ 16%, SDM1-lo and ZRP3a-lo), the volume data (porosity reduction) show a concave-up porosity change vs. effective stress trend with increasing mean effective stress above $29$ MPa, at an axial strain rate of ${10}^{-6} \, {\mathrm{s}}^{-1}$ (i.e. dilation, see Fig. 2d). This type of volumetric behaviour is typically inferred to be due to intergranular sliding leading to dilation (Pijnenburg et al. 2019a; Wong et al. 1997). At the same time, the mechanical data suggest material weakening with decreasing axial strain rate. If intergranular sliding were controlling deformation, this would suggest that this mechanism would become easier (i.e. less stress required to induce slip) with slower deformation rate, which is in line with the velocity-strengthening behaviour observed for Slochteren sandstone gouge under stress-pressure–temperature conditions comparable to our test conditions (Hunfeld et al. 2017).

By contrast, dilation was not observed in samples with a higher porosity (i.e. ~ 18–19%, SDM1-hi2; Fig. 2d). Since intergranular sliding is not dependent on porosity (Shinohara et al. 2024), this suggests that for the higher porosity Slochteren sandstone, rate-dependent intergranular slip likely plays a role during deformation. But, it has to be accompanied by a mechanism causing compaction (volume reduction), such as subcritical cracking (see Sect. 5.1.2).

### Micromechanics of strain increments accumulated under Uniaxial strain conditions

**Fig. 8:**
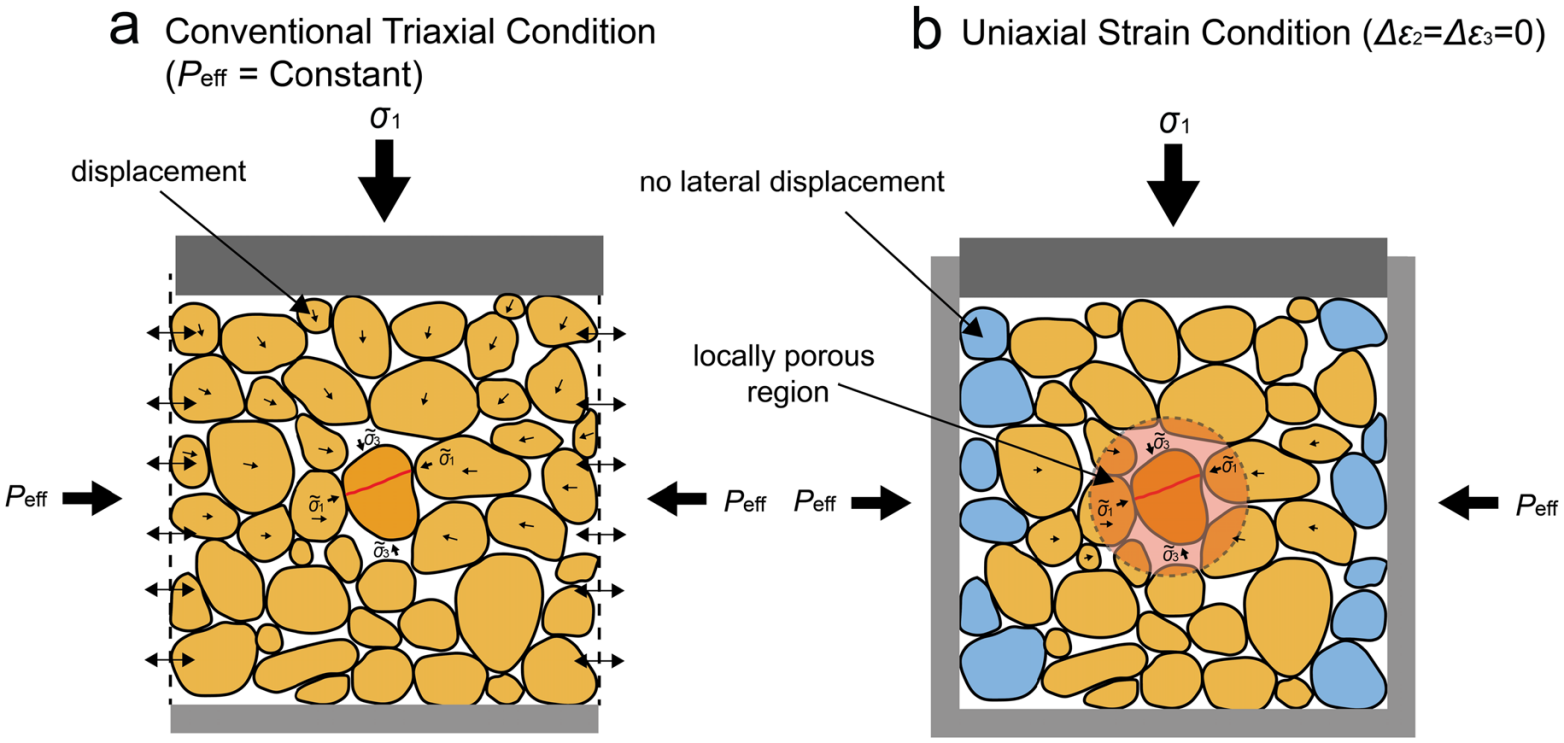
Schematic diagram illustrating the proposed micromechanical processes leading to time-dependent deformation due to stress corrosion cracking and subsequent grain rearrangement (i.e. serial grain boundary sliding—grain displacements are indicated by small, black arrows within grains) in our experiments performed under **a** conventional triaxial boundary conditions (${P}_{\text{eff}}$ = constant) and **b** uniaxial strain boundary conditions ($\Delta {\varepsilon }_{2}=\Delta {\varepsilon }_{3}=0$). The central grain shows a meridional crack (indicated in red), whose propagation is driven by the grain contact normal stress ${\widetilde{\sigma }}_{n}$. Serial grain rearrangement, which occurs subsequent to the cracking, occurs relatively easy under triaxial conditions, accommodating significant strain (Fig. 1a–c, 2a, b). By contrast, under uniaxial strain conditions, sand grains at the outer surface of the sample (i.e. at the fixed lateral boundary, indicated in blue) are inhibited from radial displacements, hence only limited grain rearrangement can occur within the whole aggregate, such as in a zone with a higher local porosity (red shaded area)

For stress corrosion cracking to be able to contribute to strain increments, serial grain rearrangement, i.e. serial intergranular sliding, is required. Under conventional triaxial boundary conditions, this serial crack growth followed by grain rearrangement through intergranular sliding can take place fairly easily, as the material is allowed to contract or dilate radially (Fig. 8a), and our triaxial experiments have demonstrated that this serial sliding likely occurs alongside parallel sliding (cf. Sect. 5.1.3).

Though under uniaxial strain conditions, where lateral movement is restricted, the effect of strain rate and pH on the axial stress–strain behaviour was negligible for the whole porosity range investigated (Figs. 2a–c, and 3c–d), the average crack density was similar to that of samples deformed under triaxial conditions (< 3% difference; Fig. 6e, i). So, under uniaxial strain conditions, decreasing strain rate or increasing pore fluid pH appeared to have a similar effect on the extent of cracking as under triaxial conditions, yet it did not lead to enhanced weakening of the material. This would imply that the contribution of individual cracks, resulting from (stress corrosion) cracking, to inelastic strain accumulated under uniaxial strain conditions is reduced compared to triaxial conditions.

Despite the absence of a noticeable rate or pH effect on the axial stress–strain behaviour under uniaxial strain conditions, we observed that the total, and rate of, increase in confining pressure, required to inhibit the sample from dilating, was reduced in a higher pH environment (i.e. 8–10 MPa ${P}_{\text{c}}$-increase at pH 7, compared to a 4–6 MPa ${P}_{\text{c}}$-increase at pH 12.3, by the end of the experiment). This effect on confining pressure was observed for both higher ($\phi =$ 18.5–18.8%) and lower porosity ($\phi =$ 14.6–15.0%) Slochteren sandstone (Fig. 3c, d, respectively), though it is more evident for the more porous material. Likewise, decreasing axial strain rate seems to also reduce the increase in confining pressure required to maintain zero-lateral strain boundary conditions, although to a much lesser extent than the observed pH-effect (i.e. the total ${P}_{\text{c}}$-increase over the entire experiment, for all porosities, is about 1–2 MPa lower at a strain rate of 10^–8^ s^−1^ than at a strain rate of 10^–6^ s^−1^; see Fig. 2a–c).

For stress corrosion cracking, coupled with serial grain boundary sliding, to explain the evolution of confining pressure under uniaxial strain conditions, while the axial stress–strain behaviour is notably independent on pore fluid pH and strain rate, locally some degree of freedom is required. Based on our microstructural observations, we propose that stress corrosion cracking, and subsequent rearrangement of the surrounding grains (i.e. grain boundary sliding), in regions with a higher local porosity (i.e. local heterogeneous porosity distribution; Fig. 6b, d, f) could be responsible for the observed stress–strain behaviour in our uniaxial strain experiments (Fig. 8b). In such locally porous regions, framework grains may not be surrounded by other load-bearing grains in all directions, meaning that they are likely not confined in at least one direction (e.g. see bottom of Fig. 5b, where several quartz grains are surrounded by unsupporting, pore-filling clay on one side). Within such an unconfined grain, crack propagation can occur at grain contact stresses smaller than those required to propagate a crack in a confined grain (Kendall 1978). Furthermore, local contact stresses may be higher in regions with a more heterogeneous porosity distribution (Shinohara et al. 2025). This implies that grains in locally more porous regions could be more susceptible to breakage, compared to regions with a more homogeneous porosity distribution. Driven by grain contact normal stress ${\widetilde{\sigma }}_{\mathrm{n}}$ (Kendall 1978; Pijnenburg and Spiers 2020), the type of cracks forming in such unconfined grains within locally more porous regions can be approximated by unconfined meridional cracks, either propagating critically or subcritically. Depending on the orientation of the contact normal stress in the locally more porous zone, we can consider two end-member cases, namely near-horizontally formed cracks and near-vertically formed cracks.

Let’s consider a centrally located grain in a zone with locally a higher porosity (see red shaded area in Fig. 8b). For near-horizontal cracks to form, the grain should be unconfined in the axial (vertical) direction and the only contact with other grains is in the horizontal direction (see Fig. 8b for a schematic). The grain contact normal stress ${\widetilde{\sigma }}_{\mathrm{n}}$, driving (subcritical) cracking, is then oriented in the horizontal direction with its magnitude approximately proportional to the effective confining pressure (i.e. ${\widetilde{\sigma }}_{\text{n}}\sim \alpha {P}_{\text{eff}}$, where $\alpha$ is a stress enhancement factor, which typically amounts to a factor of 3 for a porosity of 20% (Pijnenburg et al. 2019a). Once the horizontal crack has extended through the grain, the cracked grain can either open-up tangentially and/or the grain fragments can slide past each other along the crack surface to accommodate strain and redistribute the local stress. Given the unconfined condition in the axial direction, opening of the grain parallel to the loading direction is likely easiest, and since the grain fragments will be moving into the available pore space, these microdisplacements (of order 0.1–1 µm) will likely not affect axial stress and strain significantly. Subsequently, these small grain fragment movements offer space for the rearrangement of surrounding grains. Though, space available for rearrangement is limited, the opening of the cracked grain gives sufficient room for the surrounding, intact grains to rearrange around the cracked grain, thereby partially filling the available pore space around the cracked grain. On the whole, this results in less laterally outward movement, so the confining pressure needs to increase less to maintain a zero-lateral strain boundary (see blue grains in Fig. 8b) than if the central grain had not fractured.

On the other hand, for a near-vertically stressed grain, being unconfined in the horizontal direction, ${\widetilde{\sigma }}_{\mathrm{n}}$ is defined as $\alpha ({\sigma }_{1}-{P}_{\mathrm{p}})$, meaning the contact normal stress is higher than in the case for near-horizontally stressed grains (${\widetilde{\sigma }}_{\mathrm{n}}$~ $\alpha {P}_{\mathrm{eff}}$). Hence, the stress required to induce near-vertical cracking is likely achieved easier than the stress required for near-horizontal cracking, as the vertical stress (${\sigma }_{1}$) increases continuously in the experiment.

In addition, the near-horizontally and near-vertically stressed grains also each have a different rate- or time-dependence. Following Hertzian contact theory (Hertz 1881), the grain contact normal stress ${\widetilde{\sigma }}_{\mathrm{n}}$ is proportional to tensile stress driving crack propagation, hence also to the mode I stress intensity factor ${K}_{\text{I}}$ (e.g. Zhang et al. 1990), i.e. ${\widetilde{\sigma }}_{\mathrm{n}}\propto {K}_{\text{I}}$. Since the crack velocity $v$ is proportional to ${K}_{\text{I}}$, to the power of the so-called stress corrosion crack growth index $m$, i.e. $v\propto {K}_{\text{I}}^{m}$, the crack velocity is related to the grain contact normal stress as $v\propto {\widetilde{\sigma }}_{\mathrm{n}}^{m}$, and $m$ ~12 for wet quartz (Atkinson 1979; Atkinson and Meredith 1981). This means that at differential stress of 30 MPa (i.e. (${\sigma }_{1}$–P_p_) = 59 MPa and ${P}_{\text{eff}}$ = 29 MPa), crack propagation in the vertical direction is estimated to be ~ 3 orders of magnitudes faster than in the horizontal direction. When the material is deformed at a rate at which near-horizontal SCC is the rate-controlling process, near-vertical SCC are fast enough to be virtually time-independent process. Therefore, any contribution of near-vertical cracks to stress–strain behaviour is virtually insensitive to pore fluid pH under uniaxial strain boundary conditions, as observed in our experiments. Since our microstructural analysis showed that boundary conditions (uniaxial strain vs triaxial) do not significantly affect crack density (< 3%) and orientation distribution (Fig. 6e, i), we infer that the increased number of near-horizontal cracks at pH 12.3 (Fig. 6h), and the comparable number of near-vertical cracks observed in pH 7 and pH 12.3 environments (Fig. 6h), under triaxial boundary conditions, are likely representative for uniaxial strain conditions as well. This means that the above theoretical considerations are consistent with our experimental and microstructural observations.

In summary, our analyses suggest that, in triaxial experiments on porous Slochteren sandstone, serial stress corrosion cracking and grain boundary sliding, and independent (parallel) grain boundary sliding are likely mechanisms controlling the observed time-dependent strain. Under uniaxial strain condition, i.e. more similar to in-situ boundary conditions, serially coupled stress corrosion cracking and grain boundary sliding, without independent grain boundary sliding, is a likely mechanism controlling the observed time-dependent stress–strain behaviour. However, given the zero-lateral strain boundary conditions, these coupled processes play a more limited role in altering stress–strain behaviour, compared to the mechanisms operating under triaxial boundary conditions.

### Ongoing surface subsidence and induced seismicity in the Groningen reservoir

In the context of fluid production from sandstone reservoirs in a normal faulting regime (i.e. vertical stress ${\sigma }_{v}={\sigma }_{1}$, and ${\sigma }_{2}$ and ${\sigma }_{3}$ are horizontal), such as in the Groningen gas field, time-dependent inelastic deformation affects the evolution of ${\sigma }_{3}^{\text{eff}}$ (= ${P}_{\text{eff}}$ in our experiments; Pijnenburg et al. 2019a). Previous studies have already shown that during production, time-independent, inelastic reservoir compaction, resulting from deformation of intergranular clay films, has led to an increase in the horizontal effective stress, particularly in highly porous sandstone, such as prevalent in the seismogenic centre of the field (Pijnenburg et al. 2019a). Including inelastic compaction in predictions of the horizontal stress leads to substantially larger ${\sigma }_{3}$-values than predicted solely assuming poroelastic compaction (Pijnenburg et al. 2019a), which is often done in geomechanical modelling of compaction of the gas field (Bourne and Oates 2020; Dempsey and Suckale 2017; Smith et al. 2019; Van Eijs et al. 2006; Zbinden et al. 2017). Now that gas production is stopped, our experiments have shown that some minor to moderate ongoing compaction can be expected (up to 10%; Hol et al. 2015; Shinohara et al. 2024), due to stress corrosion cracking coupled with grain rearrangement (Fig. 8b). This inelastic compaction will also lead to an increase in the horizontal effective stress, but for an equivalent amount of vertical strain, the increase in ${\sigma }_{3}^{\text{eff}}$ will be less than seen during the production phase (i.e. when intergranular clay film compaction controls inelastic deformation). Furthermore, the bulk of the reservoir rock, away from faults, likely is under uniaxial strain boundary conditions, whereas reservoir rock in the immediate vicinity of a fault may have the freedom to move laterally, due to the stress–strain perturbations caused by the adjacent faults. This will further impact reservoir deformation across faults, as well as the horizontal stress development. Since the in-situ state of stress controls the criticality of the faults (Buijze et al. 2019, 2017; Mulders 2003; Nagelhout and Roest 1997; Orlic and Wassing 2013; Roest and Kuilman 1994; Van Eijs et al. 2006), this study demonstrates the importance of including the real, grain-scale deformation mechanisms, accurately accounting for their various inelastic contributions during different stages of the field lifetime and the boundary conditions, in models used to assess reservoir compaction, in-situ stress evolution and its relationship to induced seismicity. Our data give new constraints on the deformation mechanisms operating at reservoir relevant boundary, pressure, temperature, and strain rate conditions in the context of surface subsidence and associated induced seismicity due to gas extraction in the Groningen gas field and beyond.

## Conclusions

In this study, we identified and obtained quantitative constraints on the microphysical mechanisms accommodating time-dependent inelastic deformation in Slochteren sandstone, obtained from the seismogenic centre of the Groningen gas field in the Netherlands. We performed constant strain rate experiments (${10}^{-6} \, {\text{s}}^{-1}$ or ${10}^{-8} \, {\text{s}}^{-1}$; ${P}_{\text{eff}}$ = 29 MPa) on Slochteren sandstone samples with a porosity ranging from 14.6 to 18.9%, under both conventional triaxial and uniaxial strain (zero-lateral displacement) conditions at varying pore fluid pH (7 and 12.3) and temperature (20 and 100 $^\circ{\rm C}$). The experimental work was complemented by a quantitative microstructural comparison of undeformed and deformed samples, to further constrain the deformation mechanisms causing compaction. We concluded the following points:

1. Under triaxial boundary conditions, constant strain rate experiments performed at $T=20^\circ{\rm C}$ showed a lowering of the stress–strain curves with decreasing strain rate (${\dot{\varepsilon }}_{1}$ from $1.0\times {10}^{-6}$ to $1.0\times {10}^{-8} \, {\mathrm{s}}^{-1}$) at all differential stress levels. By contrast, under uniaxial strain boundary conditions, decreasing strain rate does not seem to affect the axial stress vs. axial strain behaviour, though less increase in confining pressure is required to maintain a uniaxial strain boundary condition at a slower deformation rate.
2. Systematic experiments varying pore fluid pH and temperature showed weakening of the Slochteren sandstone, i.e. indicated by lowering of the differential stress–axial strain curves, with increasing pore fluid pH from 7 to 12.3 and with increasing temperature from 20 to 100$^\circ{\rm C}$ at all differential stress levels under conventional triaxial conditions. By contrast, in the uniaxial strain experiments, virtually no weakening was observed in the axial stress–strain behaviour with increasing pH. However, the evolution of confining pressure, to ensure zero-lateral strain boundary conditions, showed less increase in ${P}_{\text{eff}}$ with increasing pH, for both lower ($\phi =$ 14.6–15.0%) and higher porosity ($\phi =$ 18.5–18.8%). This effect was also stronger in material with a higher porosity.
3. Our mechanical data was complemented by microstructural analysis of crack density and crack orientation distribution. The mechanical behaviour (strain rate, pH and temperature dependency), combined with the microstructural observations, suggest that stress corrosion cracking coupled with subsequent grain rearrangement (serial grain boundary sliding) is the main mechanism causing inelastic deformation under both triaxial and uniaxial boundary conditions. Furthermore, in the triaxial experiments, intergranular sliding may also operate in parallel, explaining the larger accumulated time-dependent strain in those experiments.
4. No evidence for pressure solution (i.e. pits with surface morphology of indenting grains and precipitation of the dissolved material) was found on the surface of the quartz plate deformed during creep experiments, where creep rates reached down to ~${10}^{-10} {\text{s}}^{-1}$ at differential stress $Q$ of 29 MPa and $T=130^\circ{\rm C}$.
5. Our results suggest that reservoir compaction will be limited after production stops. However changes in stress in the reservoir are expected, notably in response to the uniaxial strain boundary conditions pertaining in the bulk of reservoir away from faults. Close to faults, on the other hand, deformation may no longer be uniaxial strain, due to stress–strain perturbations, associated with enhanced deformation as evidenced by our triaxial experiments. Therefore, time-dependent inelastic deformation needs to be accounted for in models assessing reservoir compaction and its relationship to induced seismicity in the Groningen gas field.

## Data Availability

The mechanical and microstructural data used for this paper are available through 10.24416/UU01-ELSYBJ. Yoda data publication platform of Utrecht University.
